# Assessing the Oxidative Potential of Outdoor PM_2.5_ in Wintertime Fairbanks, Alaska

**DOI:** 10.1021/acsestair.3c00066

**Published:** 2024-02-10

**Authors:** Yuhan Yang, Michael A. Battaglia, Magesh Kumaran Mohan, Ellis S. Robinson, Peter F. DeCarlo, Kasey C. Edwards, Ting Fang, Sukriti Kapur, Manabu Shiraiwa, Meeta Cesler-Maloney, William R. Simpson, James R. Campbell, Athanasios Nenes, Jingqiu Mao, Rodney J. Weber

**Affiliations:** † School of Earth and Atmospheric Sciences, Georgia Institute of Technology, Atlanta, Georgia 30332, United States; ‡ Department of Environmental Health & Engineering, Johns Hopkins University, Baltimore, Maryland 21218, United States; § Department of Chemistry, University of California, Irvine, California 92697, United States; ∥ Geophysical Institute and Department of Chemistry & Biochemistry, University of Alaska Fairbanks, Fairbanks, Alaska 99775, United States; ⊥ Laboratory of Atmospheric Processes and their Impacts (LAPI), School of Architecture, Civil & Environmental Engineering, Ecole Polytechnique Fédérale de Lausanne, Lausanne 1015, Switzerland; # Center for Studies of Air Quality and Climate Change, Institute of Chemical Engineering Sciences, Foundation for Research and Technology Hellas, Patras 26504, Greece

**Keywords:** subarctic region, residential heating, biomass burning, fine particulate matter (PM_2.5_), oxidative potential, multivariate linear regression, transition metals, vehicle emissions

## Abstract

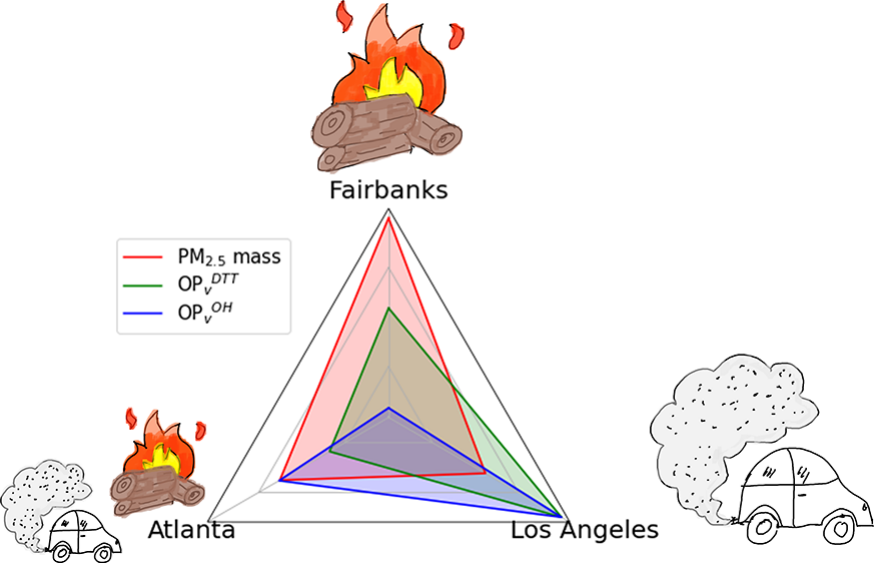

The oxidative potential (OP) of outdoor PM_2.5_ in wintertime Fairbanks, Alaska, is investigated and compared to those in wintertime Atlanta and Los Angeles. Approximately 40 filter samples collected in January–February 2022 at a Fairbanks residential site were analyzed for OP utilizing dithiothreitol-depletion (OP^DTT^) and hydroxyl-generation (OP^OH^) assays. The study-average PM_2.5_ mass concentration was 12.8 μg/m^3^, with a 1 h average maximum of 89.0 μg/m^3^. Regression analysis, correlations with source tracers, and contrast between cold and warmer events indicated that OP^DTT^ was mainly sensitive to copper, elemental carbon, and organic aerosol from residential wood burning, and OP^OH^ to iron and organic aerosol from vehicles. Despite low photochemically-driven oxidation rates, the water-soluble fraction of OP^DTT^ was unusually high at 77%, mainly from wood burning emissions. In contrast to other locations, the Fairbanks average PM_2.5_ mass concentration was higher than Atlanta and Los Angeles, whereas OP^DTT^ in Fairbanks and Atlanta were similar, and Los Angeles had the highest OP^DTT^ and OP^OH^. Site differences were observed in OP when normalized by both the volume of air sampled and the particle mass concentration, corresponding to exposure and the intrinsic health-related properties of PM_2.5_, respectively. The sensitivity of OP assays to specific aerosol components and sources can provide insights beyond the PM_2.5_ mass concentration when assessing air quality.

## Abstract 2

This study highlights the sensitivity of different oxidative potential assays to specific aerosol components and likely emission sources, offering valuable insights beyond PM_2.5_ mass concentration for air quality assessment.

## 1 Introduction

Fairbanks is a subarctic city (64.84°N latitude) in Alaska’s interior with unique wintertime meteorology and emissions that contribute to high fine particle (PM_2.5_) mass concentrations that often exceed air quality standards (e.g., 24-h average of 35 μg/m^3^). During the November to March winter (cold) season, low solar insolation leads to extremely low temperatures (January average low of ∼ −25 °C) and strong near-surface temperature inversions that limit the dispersion of surface-emitted pollutants.^1,2^ A main source for the high PM_2.5_ concentrations is residential heating with wood, which is estimated to contribute 19–80% to overall outdoor PM_2.5_ mass, although it has been decreasing in recent years.^3−7^ The sulfate mass fraction ranges from 8 to 33%, which is mainly from the combustion of sulfur-containing fuels, such as home heating oil.^2,5,8^ Other sources of PM_2.5_ are vehicle-related emissions that contribute 0–31%^4,5,7,9^ as well as minerals and salts for road traction at <5% of the mass fraction.^5,7^ Emissions and PM_2.5_ mass concentrations in Fairbanks and surrounding communities are not spatially homogeneous. The downtown area is influenced more by vehicles, while the residential neighborhoods (especially the nearby town of North Pole) have higher biomass burning emissions from residential wood heating.^10^

As a health-related metric, mass concentration does not consider variations in chemical composition, size, and physical properties, all of which are affected by sources and aging processes and all of which are expected to modulate toxicity and health effects. An alternative approach to address some of this shortcoming is to focus on chemical species that drive adverse responses, but this is challenging due to their complexity and dynamic nature. One approach of increasing interest is to quantify the aerosol oxidative potential (OP), which ideally is an integrative metric of the chemical species that can cause oxidative stress via the formation of reactive oxygen species (ROS) in cells and tissues. Organic peroxides and redox-active components, such as aromatic species and transition metals, are significant contributors to particle OP.^11^ Redox-active species may catalytically generate ROS *in vivo.*

The unique characteristics of Fairbanks wintertime PM_2.5_ may translate to correspondingly unique health impacts. The prevalence of heating from wood burning could produce especially unhealthy PM_2.5_.^12−14^ Residential wood combustion emits hazardous air pollutants, such as polycyclic aromatic hydrocarbons (PAHs) and heavy metals,^15,16^ and has been linked to disease burden and premature death in subarctic regions.^17,18^ A further unique feature of Fairbanks winter is minimal sunlight, resulting in low concentrations of oxidants (e.g., H_2_O_2_ and ·OH) and secondary species. Surface ozone levels during polluted episodes are low due to titration by combustion-emitted NO and minimal photochemical generation.^2^ Consequently, the typical photochemical aging of particles, which can enhance particle toxicity,^19−23^ is constrained. Other aging processes linked to particle toxicity,^24^ such as the solubilization of metals emitted from noncombustion sources, may be restricted by the relatively high particle pH in Fairbanks^8^ and low concentrations of organic species, like oxalate, that form soluble metal–organic complexes.^25−28^ Thus, more characterization of the health-related characteristics of PM_2.5_ in populated Arctic regions is needed.

There are several acellular assays available to measure particle OP. Most frequently used assays include the dithiothreitol (DTT) depletion assay that mimics loss of antioxidants; techniques to measure ROS directly; and measuring the production rate of oxidants, such as the measurement of hydroxyl radicals (·OH) in surrogate lung fluid (SLF).^29−34^ The various assays are sensitive to distinct panels of chemicals.^31^ OP^DTT^ responds to certain metals and organic species, such as Cu, Mn, and aromatic compounds, notably quinones.^35,36^ OP^OH^ responds to fewer organic species but preferentially to species involved in Fenton-type electron transfer reactions, especially iron. Synergistic and antagonistic interactions between metals and humic-like substances (HULIS) or quinones can also influence the OP determined by a specific assay.^37−39^ Studies have linked acellular assays to specific adverse health effects,^40−45^ or as modifiers of PM_2.5_ adverse effects.^46−52^ In some cases, OP is more strongly associated with specific health end points than PM_2.5_ mass concentration.^30,40,43,47,53^ In contrast, a few studies have not found associations between OP and health effects.^54−56^ Using a combination of acellular assays to measure OP has provided different insights than PM_2.5_ mass concentration when identifying detrimental sources and hazards for populations in different regions.^53^ A number of reviews summarize various assays, the chemical species they respond to, and their links to health outcomes.^30,31,57,58^ The DTT assay, while responsive to a broad range of chemical components, primarily corresponds to the formation of superoxide (O_2_^•–^) and does not include the generation of ·OH, an important step of the ROS cascade.^11,33^ To address this limitation, we chose the OP^DTT^ and OP^OH^ assays for this study since they may provide a more comprehensive assessment, potentially capturing a wider array of health-relevant species that might be overlooked by a single assay approach.

Air quality in populated Arctic regions is not well characterized.^59^ To address this, we investigated the levels of OP and possible health-influencing properties of Fairbanks PM_2.5_ by complementary assays during the winter high-pollution period and explored the major chemical species driving OP using multivariate linear regression (MLR). We compared these results to other urban regions, contrasting OP assays and OP to PM_2.5_ mass concentrations, to assess if Fairbanks winter PM_2.5_ is uniquely harmful.

## 2 Methods

### 2.1 PM_2.5_ Sampling

This research is part of the Alaskan Layered Pollution And Chemical Analysis (ALPACA) field campaign. Ambient outdoor PM_2.5_ samples were collected from 17 January 17, 2022 to 25 February 25, 2022, at the ALPACA House field site (64.850°N, 147.676°W) located in a residential area (Shannon Park Neighborhood) roughly 2.6 km from downtown ALPACA sites, National Core (NCore) and University of Alaska Fairbanks Community and Technical College (CTC), in Fairbanks. A total of 49 PM_2.5_ filter samples (including 7 blanks and 2 samples tested at the outset of the study, resulting in a total of 40 effective samples) were collected over the study period using a Tisch PM_2.5_ high-volume (Hi-Vol) sampler (un-denuded and with a flow rate of normally 1.13 m^3^/min), and each filter was collected over 23.5 h (10:00 am to 9:30 am next day) using pre-baked quartz filters (20.32 × 25.40 cm; Whatman QM-A quartz filter) with a filter particle collection area of 516.13 cm^2^. The collected samples were promptly sealed with pre-baked aluminum foil and stored at −20 °C until analysis.

### 2.2 Acellular Oxidative Potential Measurements

The high-volume filters were analyzed for OP by two techniques, the DTT depletion assay (OP^DTT^) for water-soluble (WS) and all (total) PM_2.5_ (this assay is not performed in synthetic lung fluid) and OH production in the synthetic lung fluid assay (OP^OH^) for total PM_2.5_. A fraction from each filter was placed in a sterile polypropylene centrifuge vial (VWR International LLC, Suwanee, GA, USA). Due to the possible nonlinear response of OP end points with extract mass concentration,^36^ the fraction of the filter and the volume of water used for extraction were determined based on the PM_2.5_ mass loading on each filter to achieve a relatively constant sample concentration of 10 μg/mL for WS and total OP^DTT^ analysis and 25 μg/mL for OP^OH^ analysis in the respective reaction vials. Filters were extracted in deionized Milli-Q water (DI, Nanopure InfinityTM ultrapure water system; resistivity > 18 MΩ/cm) via 60 min sonication (Ultrasonic Cleanser, VWR International LLC, West Chester, PA, USA).

For water-soluble (WS) analysis (OP^WS DTT^), the water extracts were further processed by filtering through a 0.45 μm poly(tetrafluoroethylene) (PTFE, Fisherbrand, Fisher Scientific, Hampton, NH, USA) syringe filter. For OP^total DTT^ and OP^OH^ measurements, the PM extracts were not filtered, and the filter punch was left in the extracts throughout the OP analysis so insoluble species could be in contact with the reagents.^60^ Established protocols were used for the OP^DTT^ and OP^OH^ methods,^60−63^ with details given in the Supporting Information. Both volume (OP_v_^WS DTT^, OP_v_^total DTT^, OP_v_^OH^) and mass normalized (OP_m_^WS DTT^, OP_m_^total DTT^, OP_m_^OH^) results are discussed, where volume-normalized OP is normalized by volume of air sampled and applicable to exposure, and mass-normalized OP is normalized to the mass of PM_2.5_ and representative of an intrinsic health-relevant property of the PM_2.5_.

### 2.3 Aerosol Mass Concentration and Composition Measurements

Hourly PM_2.5_ mass concentration was measured by a Beta Attenuation Monitor (BAM) at the NCore monitoring site, which is in central Fairbanks and 2.6 km from the House site and operated by the Alaska Department of Environmental Conservation for the U.S. Environmental Protection Agency. Nonrefractory PM_1_ composition (NH_4_^+^, NO_3_^–^, SO_4_^2–^, Cl^–^, and organic aerosol (OA) including PAHs) was measured at the House site with a High-Resolution Time-of-Flight Aerosol Mass Spectrometer (HR-ToF-AMS, Aerodyne Research, Inc., USA). A three-factor positive matrix factorization (PMF) analysis of the mass spectra yielded the factors of hydrocarbon-like OA (HOA), biomass-burning OA (BBOA), and an additional primary organic aerosol factor (POA2). HOA and BBOA are standard factors derived from AMS mass spectra. The remaining mass was apportioned to a factor named “POA2”, which does not resemble the mass spectrum of any canonical secondary OA (SOA) or oxidized OA (OOA). Instead, it has characteristics of a variety of primary OA spectra (e.g., cooking, vehicles, and others) (see Supporting Information). Due to the minimal sunlight (4-6 h/day) and relatively short pollution residence time (median of 2.1 h)^64^ in wintertime Fairbanks, the photochemical aging of particles was limited, resulting in low levels of SOA, as resolved by the PMF solution, in contrast to most other studies. The same filters used for the OP analysis were also utilized for other analyses. Organic carbon (OC) and elemental carbon (EC) were determined by the thermal-optical-transmittance method following the NIOSH 5040 analysis protocol.^65^ Concentrations of elements were determined by inductively coupled plasma mass spectrometry (ICP-MS). This included total metals, such as magnesium, aluminum, potassium, manganese, iron, copper, zinc, and lead, and water-soluble metals. The latter were prepared by extracting filters in water followed by 0.45 μm pore PTFE filtration then ICP-MS analysis.^66^ Both water and methanol-soluble brown carbon (WS and MS BrC, respectively, i.e., light absorption at 365 nm wavelength) based on separate filter punches were determined with UV-Vis spectrophotometry.^67^ Different time-based data were averaged to the filter sampling time (24 h) for comparisons. Additional methodological details can be found in Supporting Information.

### 2.4 Multivariate Regression

MLR was used to quantify the specific PM_2.5_ species contributing to the measured PM_2.5_ OP, enabling comparisons in toxicity between different classes of species (i.e., organic vs metal), contrasting contributions between different sources to a given OP assay, as well as investigation of differences between the OP assays. Stepwise regression was applied for variable selection. Unstandardized and standardized models were employed, with the former fitting the models using the raw data, and the latter rescaling the data using a linear transformation to achieve a mean of 0 and variance of 1 for all variables (for more details, see the Supporting Information and the following discussion). Explanatory variables were selected for the model from the following list: OA types, including BBOA, HOA and POA2, and PAHs (which could be a subset of the BBOA, HOA, and POA2), WS BrC, MS BrC, EC, and four total and water-soluble metals (Mn, Fe, Cu, and Zn). Inorganic ions (e.g., NH_4_^+^, NO_3_^–^, SO_4_^2–^, and Cl^–^) resulted in poorer fits and were not included, which is consistent with other studies showing they often do not have a significant direct impact on aerosol OP.^38,53,68^ Before the regression analysis, extreme outliers were removed (see Supporting Information). The correlation between PM components and various OPs was assessed, and only those components that showed a correlation coefficient (*r*) greater than 0.5 and a *p* value less than 0.01 were selected as independent variables for the regression models. Strong collinearities were observed between several PM components, including BBOA, POA2, WS BrC, MS BrC, and WS Fe, and between EC and HOA (Table S2). One species in each set was included in a single model, and the MLR model with a greater coefficient of determination and lower mean squared error (MSE) was selected. The identified sources are largely independent of the variables included in the regression models. The MLR model here assumed that predictor responses are exclusively additive, which may not hold universally, and synergistic or antagonistic interactions among predictor variables could occur.^33,37,69,70^ Regressions considering interaction terms were also performed,^37^ which did not provide better fits for the DTT assay, and only slightly better fits for the OH assay. The suite of results is tabulated along with more method details in the Supporting Information.

## 3 Results

### 3.1 PM_2.5_ Characteristics during the Study

**Figure 1:**
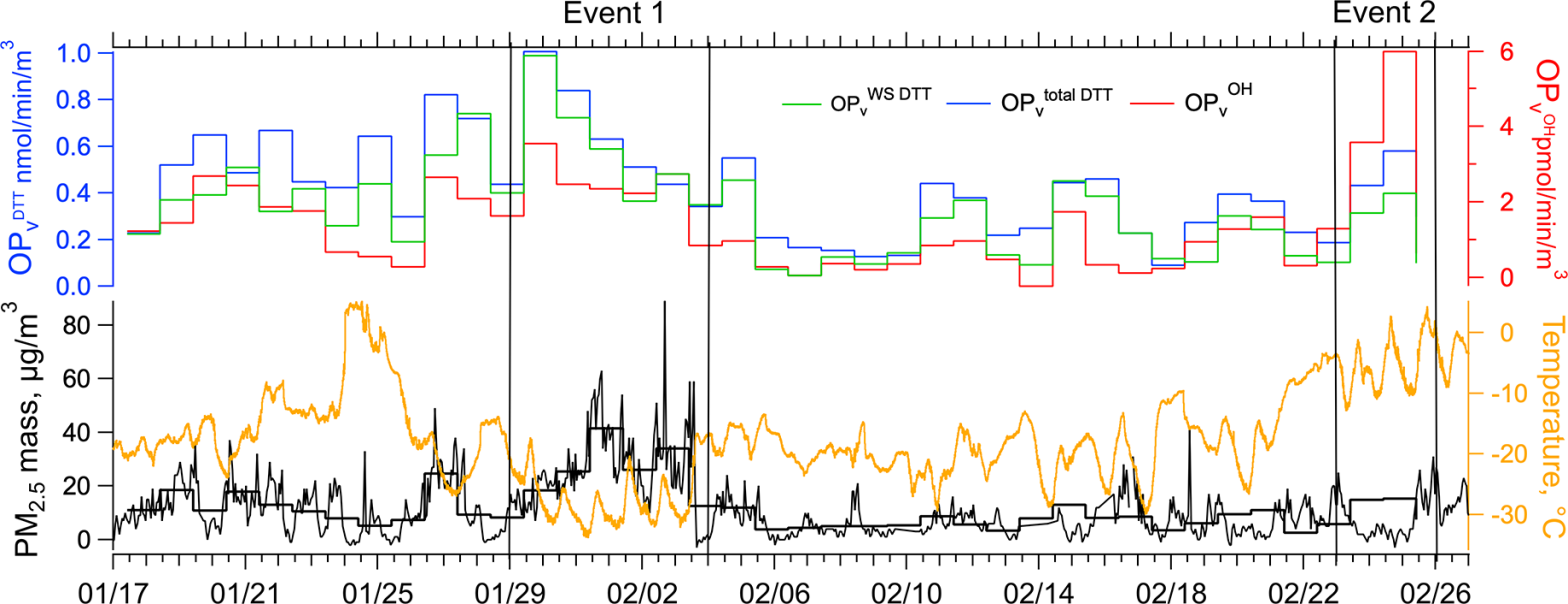
PM_2.5_ mass concentration (1 and 24-h averages), temperature, and nominally 24-h average OP_v_^WS DTT^, OP_v_^total DTT^, and OP_v_^OH^ during the ALPACA study period. Two pollution events are identified as Event 1 (1/29/22–2/4/22) and Event 2 (2/23/22–2/26/22).

Figure 1 shows hourly and 24 h averaged PM_2.5_ mass concentration measured at the NCore monitoring site and temperature 3 m above ground level at the CTC site (roughly 580 m from NCore site). Both the PM_2.5_ mass and temperature (*T*) showed significant variability. For the study period, the mean (± standard deviation based on 1 h data) PM_2.5_ mass was 12.8 ± 11.1 μg/m^3^, and the temperature was −17.5 ± 7.8 °C. PM_2.5_ mass concentration at the House site was not measured, so it was determined by merging the AMS-measured species (Cl^–^, NO_3_^–^, SO_4_^2–^, NH_4_^+^, OA) to the filter sampling times and summing with EC and metals from the filters. Some differences are expected (Figure S1) since the data are from two different locations.^10^ The AMS measures nonrefractory PM_1_, not PM_2.5_, and there are missing chemical components that contribute to mass. The largest discrepancy was during the main pollution event (Figure 1, Event 1) when PM_2.5_ mass concentrations were highest (Figure S1b). Despite these issues, the calculated PM_2.5_ at the House site agrees well with NCore PM_2.5_ (slope = 1.04, intercept = 2.07 μg/m^3^, and *r*^2^ = 0.70; see Figure S1a). The estimated PM_2.5_ mass concentration at the House site was used in subsequent analysis.

**Figure 2:**
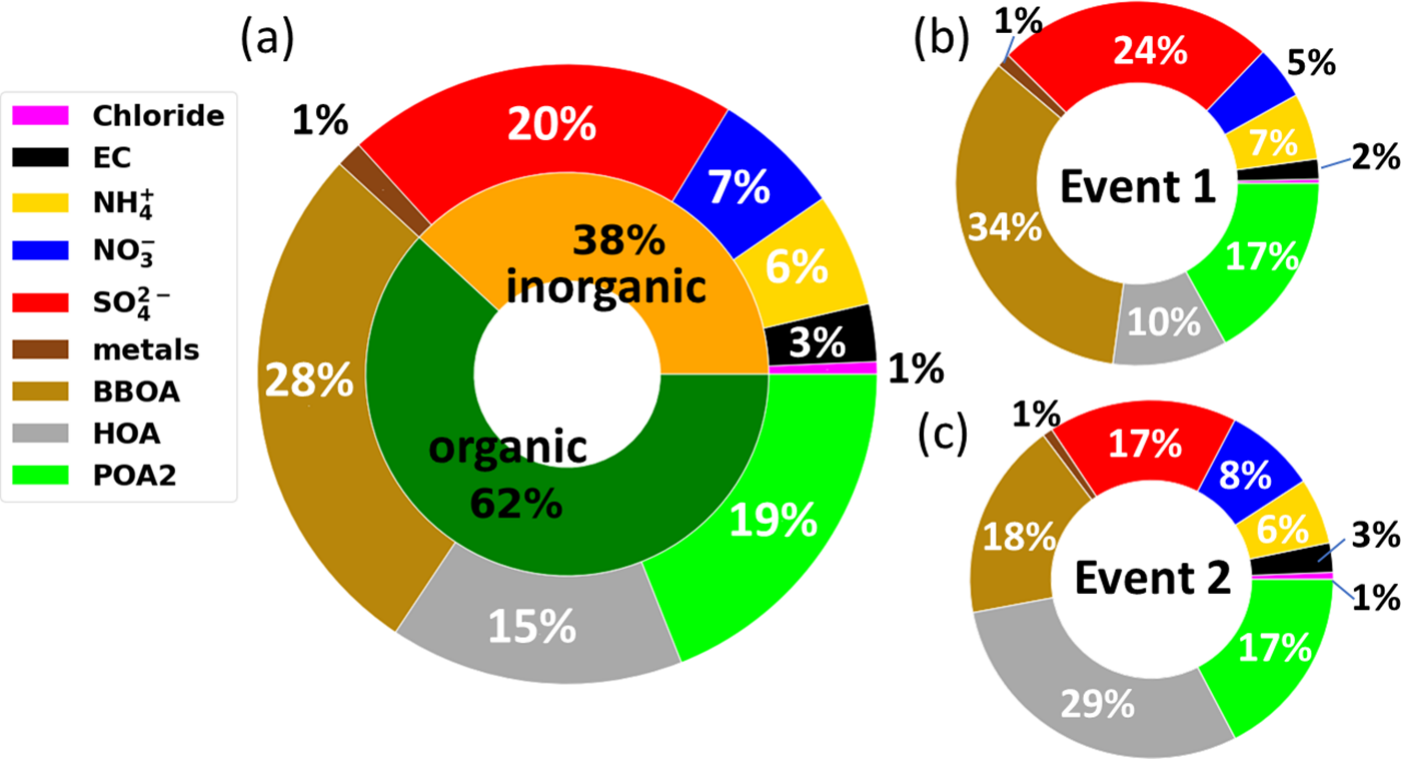
(a) Average PM_2.5_ composition during the whole study period (1/17/2022–2/25/2022), (b) Event 1 (1/29/22–2/4/22), and (c) Event 2 (2/23/22–2/26/22) identified in Figure 1.

**Figure 3:**
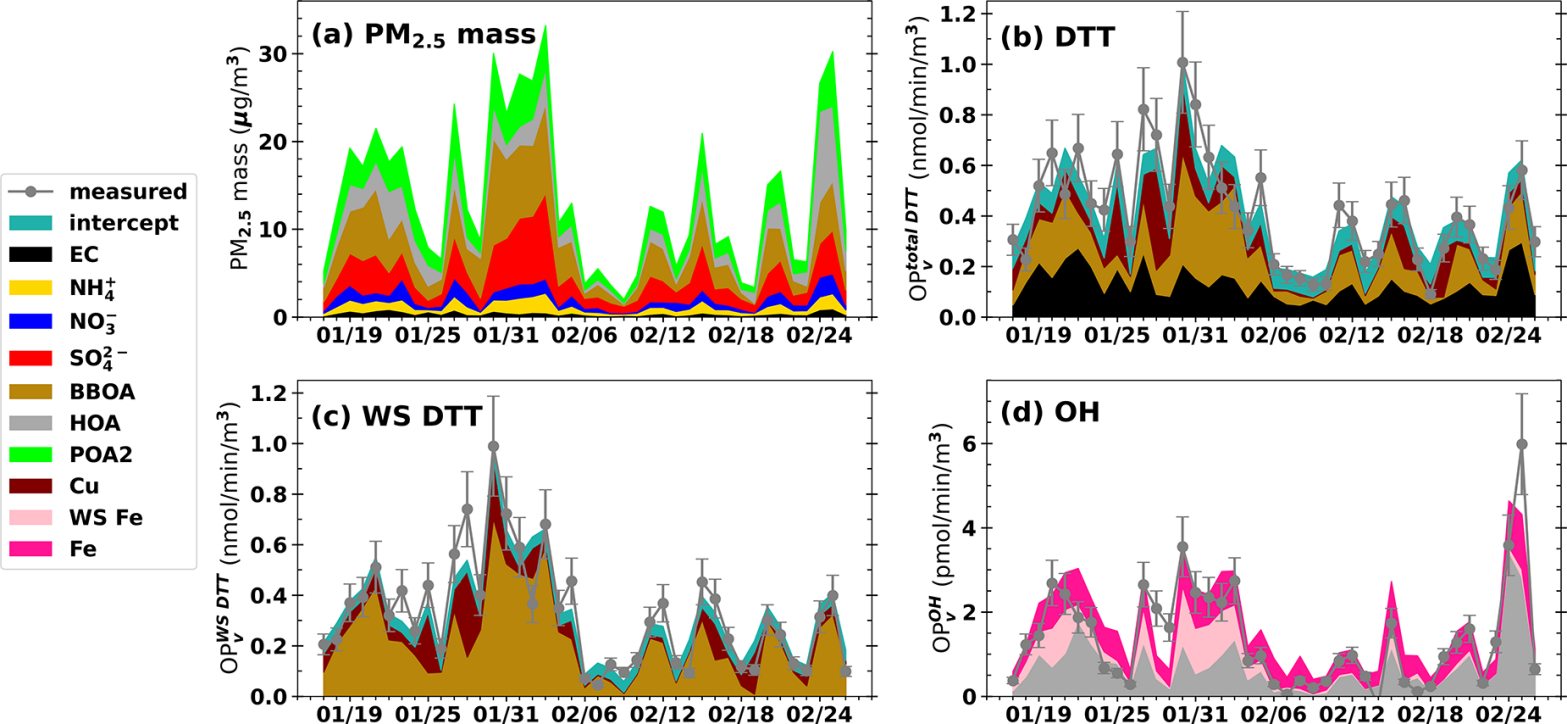
Time series of the contributions of PM components to (a) PM_2.5_ mass concentration, (b) OP_v_^total DTT^, (c) OP_v_^WS DTT^, and (d) OP_v_^OH^. The various OPs were determined from the time series of PM_2.5_ components concentration (units in μg/m^3^) multiplied by the regression coefficients (units in nmol/min/μg for OP^DTT^ and pmol/min/μg for OP^OH^) for the unstandardized model (eqs 1–3). The *x*-axes are dates in Month/Day for 2022.

Figure 2a shows the study average PM_2.5_ composition, while Figure 3a shows the time series of the PM_2.5_ composition. OA was the dominant component accounting for a mass fraction of ∼62% of the wintertime PM. Among the OA, 45% was BBOA, 31% was identified as POA2, and 25% was identified as HOA. Sulfate was the second largest component accounting for a mass fraction of 20% of PM_2.5_. EC was 3% and the sum of all measured metals (elemental mass) was a small mass fraction at 1.4%.

### 3.2 Oxidative Potential

The time series of OP measured in Fairbanks is also shown in Figure 1. The mean OP_v_^total DTT^ was 0.42 nmol/min/m^3^, while the mean for OP_v_^OH^ was 1.40 pmol/min/m^3^ (Table S1). Typically, OP_v_^DTT^ measurements from various emission sources (including burning of various fuels, traffic, and secondary formation), or ambient conditions (including both urban and rural environments), in different regions of the United States range from 0.04 to 0.66 nmol/min/m^3^, making Fairbanks above average.^38,60,61,63,68,71,72^ Studies in California and the Midwestern U.S. reported OP_v_^OH^ in SLF values of 0.253 to 7.884 pmol/min/m^3^,^71,73,74^ showing that Fairbanks OP_v_^OH^ was somewhat below the average of these studies. The mean of OP_m_^total DTT^ in Fairbanks was 0.035 nmol/min/μg, which is within the range of 0.001 to approximately 0.2 nmol/min/μg reported in other studies.^30,34^ The mean OP_m_^OH^ in Fairbanks was 0.119 pmol/min/μg, towards the bottom of the range of 0.092 to 0.967 pmol/min/μg from other observations.^63,74,75^ Based on these study-average data, Fairbanks OP levels were not exceptional. For conditions during the most polluted period (Event 1) when 1-h PM_2.5_ reached a study maximum of 89 μg/m^3^ (40.25 μg/m^3^ for 24-h average, Table S1), the 24-h average OP_v_^total DTT^ and OP_v_^OH^ values were higher than the Fairbanks mean by a factor of about 2 (Event 1 in Figure 1).

Analysis of correlations can provide insights into possible relationships between the PM_2.5_ mass concentration and OP, as well as highlight contrasts between the OP methods. However, in Fairbanks, high correlations can also be driven by synchronized temporal variability in various air quality parameters, which substantially fluctuates due to dramatic changes in the strength of the temperature inversions. Relative differences in the degree of correlations are most useful.

Pearson’s correlation coefficients (*r*) between PM_2.5_ mass concentration and the OP assays on a per volume air basis are shown above the diagonal in Table 1. The highest correlation was observed between the OP_v_^OH^ and PM_2.5_ mass concentration (*r* = 0.85), whereas for the OP^total DTT^ the correlation was 0.7. Given that volume-normalized OP is relevant to exposures, for this study, the OH-production assay would give a somewhat similar view as PM_2.5_ mass concentration as a health hazard since about 72% (*r*^2^) of the variability in OP_v_^OH^ follows the variability in PM_2.5_ mass concentration, but only about half (*r*^2^ = 0.5) for OP_v_^total DTT^. Correlation coefficients for mass-normalized OP, data below the diagonal in Table 1, showed that in all cases the various OP_m_ values were negatively correlated with PM_2.5_ mass concentration, demonstrating that some PM components that contributed to PM_2.5_ mass did not significantly contribute to the responses by these assays. The overall interpretation is that there were components of PM_2.5_ that contributed to the various OP values and were temporally correlated with PM_2.5_ mass, but they contributed minor amounts to the overall PM_2.5_ mass concentration.

**Table 1:** Pearson’s Correlation (*r*) between PM_2.5_ Mass Concentration and Oxidative Potential (OP)^a^

	PM_2.5_	OP^total DTT^	OP^WS DTT^	OP^OH^
PM_2.5_	1	0.70^b^	0.73^b^	0.85^b^
OP^total DTT^	–0.56^b^	1	0.89^b^	0.66^b^
OP^WS DTT^	–0.35^c^	0.70^b^	1	0.64^b^
OP^OH^	–0.32	0.57^b^	0.34	1

a Correlations for volume-normalized OP are above the diagonal and below for mass-normalized OP. Data include all PM_2.5_ measured in Fairbanks, based on 24-h averages.

b *p*-value < 0.001.

c *p*-value < 0.05.

For correlations between the OP assays, the volume-normalized OP measurements had moderate correlations (ranging from 0.64 to 0.89). OP^total DTT^ and OP^WS DTT^ showed the strongest relationship (*r* = 0.89 for air volume-normalized and *r* = 0.70 for PM_2.5_ mass-normalized; see Table 1 and Figure S2 for the regression results). This is expected since OP^WS DTT^ is a significant subset of OP^total DTT^; on average (± standard deviation), the ratio of OP_v_^WS DTT^ to OP_v_^total DTT^ was 77 ± 27%. The correlation between OP_v_^WS DTT^ or OP_v_^total DTT^ with OP_v_^OH^ was between 0.64 and 0.66 (*r*^2^ = 41% and 44%), meaning less than half of the DTT and OH assay’s variability was related. These two assays were sensitive to different PM chemical components, demonstrating their complementary nature.

### 3.3 Multivariate Linear Regression Analysis

The unstandardized and standardized MLR models predicted the measured OP variability well; for both regressions, the overall coefficients of determination (*r*^2^) between the modeled and measured results were greater than 0.7. High correlation between many species in Fairbanks due to meteorology accounts for some of the good model performance based on coefficients of determination. The intercept of the models, which is the residual that the model could not represent, accounted for 13–22% of the mean OP for unstandardized and 1.5–7.1% for standardized MLRs (see regression equations below and in Supporting Information).

Strong collinearities were observed between several PM components, including PAH, BBOA, POA2, WS BrC, MS BrC, and WS Fe (Table S2), and between EC and HOA. All these species could largely represent emissions from combustion sources. Amongst the highly correlated species, only one was included in a single model, and the MLR models with the best fits (greater coefficient of determination, lower mean squared error, MSE, and lower intercept) are shown below. Other regression results are given in eqs S1–S32 for reference. All regression results produce a similar interpretation of the sources and chemical species affecting the measured OP.

The overall results from the regression analysis are shown in Figure 3, which gives the time series of the chemical components contributing to the various OP values for the study period. Detailed MLR results are discussed next.

Examples of the unstandardized model results are summarized in eqs 1, 2, and 3. The regression coefficients (units of nmol/min/μg for the DTT assay and pmol/min/μg for the OH assay) indicate the relative intrinsic importance of various species when applied to a specific OP assay. The coefficient associated with each independent variable represents the change in OP_v_ (units of nmol/min/m^3^ for DTT assay and pmol/min/m^3^ for OH assay) per unit increase in the concentration of that variable by 1 μg/m^3^. The results are
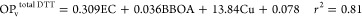
1
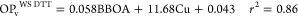
2
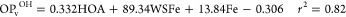
3

Examples of the standardized model results are given in eqs 4, 5, and 6, and in this case, the unitless regression coefficients are the relative importance of various independent variables on OP considering their actual ambient air mass concentration, where a coefficient represents the change in OP_v_ (unit of 1) per unit increase in a specific PM component concentration by 1 standard deviation. These coefficients then assess the relative significance of different OP accounting for actual concentrations (exposure).
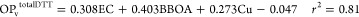
4
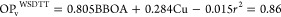
5
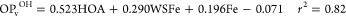
6

Equations 1 and 4 show that EC, BBOA, and Cu variability can be used to predict the observed OP_v_^total DTT^. For an equal change in mass concentration, Cu had a much greater effect on OP_v_^total DTT^ than did EC and BBOA (eq 1). The average concentrations of EC and BBOA were 0.437 and 3.85 μg/m^3^, respectively, whereas the average Cu concentration was 5 ng/m^3^ (Table S3). Thus, despite OP^total DTT^ being more sensitive to Cu, EC and BBOA had a greater impact in terms of exposure since the standardized regression coefficient for EC was 0.308 and 0.403 for BBOA compared to 0.273 for Cu (eq 4 and Figure 3b).

A similar type of analysis can be done for the OP_v_^WS DTT^. In this case, BBOA and Cu were selected by the MLR model as contributors. In all the assays, we expect metal ions rather than insoluble metals to drive OP, for example, Cu ions in driving OP_v_^total DTT^ and OP_v_^WS DTT^, although the MLR selected total Cu. Total Cu can encompass surface-active Cu species, potentially tightly adsorbed onto the surface of an insoluble yet reactive particle, like soot, which remains unextractable by water and contributes to the OP^DTT^ response; alternatively, the omission of WS Cu as a factor in the MLR model could be due to its strong correlation with WS BrC and MS BrC (Table S2), which suggests a biomass burning source that is already accounted for by the inclusion of the BBOA factor in the models. Again, OP was more sensitive to Cu than the bulk OA species that comprises BBOA; corresponding coefficients in eq 2 are 0.058 nmol/min/μg for BBOA vs 11.68 nmol/min/μg for Cu. But again, concentrations of BBOA were much higher than those of Cu, so BBOA had a larger influence on OP_v_^WS DTT^ during the study considering the exposure (eq 5 and Figure 3c).

Both OP^total DTT^ and OP^WS DTT^ were measured using the same assay, but OP^total DTT^ includes insoluble species (about 23% on average). It is noteworthy that BBOA and Cu were significant contributors to both; however, EC was only selected by the MLR in OP_v_^total DTT^ not in OP_v_^WS DTT^, which suggests that aromatic species played a role in both OP_v_^WS DTT^ and OP_v_^total DTT^ with the added contribution of surface-bound aromatic species associated with insoluble EC.^76,77^ Other combinations of variables give similar overall results when using a different set of highly correlated variables. Most noteworthy is that EC and BBOA can be replaced by AMS-determined PAHs in the MLR for OP^total DTT^. For OP_v_^WS DTT^, BBOA can be replaced by BrC with a similar model fitting performance (see eqs S1–S9 and S15–S23). There is a noteworthy consistency between the standardized MLR of OP^DTT^ in this study and that described by Gao et al. (2020) for Atlanta using the same regression method,^38^ where 81% of the OP_v_^total DTT^ in the Atlanta winter season was attributed to BrC, 13% to EC, and 20% to Cu, implying that some aromatic species, such as quinones, emitted from biomass combustions play a significant role of in OP^DTT^ in both cities, followed by elemental carbon and copper.

For OP_v_^OH^, HOA, WS Fe, and Fe were the most significant contributors. In Fairbanks, WS Fe represented only a minor fraction of the total Fe (study average WS Fe/total Fe = 0.07 ± 0.07) and appeared to originate from distinct sources, as discussed later. Therefore, while WS Fe and Fe were not entirely independent, both were deemed significant contributors to the OP_v_^OH^. For OP_m_^OH^ total Fe had much more of an effect than HOA, and WS Fe had even more (eq 3). The OH assay is known to be very sensitive to Fenton reactions^74,78^ that involve Fe ions, which are likely a larger fraction of the WS Fe compared to total Fe, since total Fe may contain solid unreactive species, such as iron-oxides. For exposures, the HOA component of PM_2.5_ was also important based on the standardized regression for OP_v_^OH^, since the average concentration of HOA was almost 50 times higher than that of Fe (2190 ng/m^3^ vs 44 ng/m^3^). Thus, considering the concentrations, HOA had a similar effect as the combined WS Fe and total Fe (eq 6 and Figure 3d).

These results are consistent with other studies of species that influence the various assays. From this analysis, certain organic species and transition metals were key contributors to Fairbanks PM_2.5_ oxidative potential in terms of actual exposure to outdoor air.^30,79^

Correlations (summarized in Table S2) point to sources of the PM_2.5_ species that contributed to the different OP assays. BBOA, aromatic-containing compounds (PAHs serves as a tracer), and BrC exhibited strong correlations with each other (*r* > 0.85), consistent with a common wood smoke source. HOA was mostly correlated with CO and EC (*r* ≈ 0.85), both CO and EC are traffic emission tracers but also emitted by wood burning. HOA showed a weaker correlation with BBOA, PAHs, or BrC (*r* ≈ 0.5). In other locations, HOA has been associated with traffic-related emissions.^32,73,80^ Total Fe had similar correlations with CO and EC as HOA did with these species (Fe and CO *r* = 0.86, Fe and EC *r* = 0.80) and was correlated with HOA (*r* = 0.77), and Fe has been associated with traffic-related emissions in prior research.^81,82^ In contrast, WS Fe had a very high correlation with BBOA (*r* = 0.9), PAHs (*r* = 0.83), and BrC (*r* = 0.89), indicating it was mainly from wood burning and largely a different source than total Fe. This is unique to Fairbanks and may result from a lack of conversion of total Fe to WS Fe in this environment due to low concentrations of organic species that could form metal–organic complexes (e.g., oxalate), or too high a particle pH (pH of 3–5), or both. Cu did not have strong correlations with specific species but showed some correlation with EC (*r* = 0.26) and BrC (*r* = 0.34). Therefore, we conclude that HOA and Fe were mainly from vehicle emissions, while WS Fe, PAHs, and BBOA were mainly from heating with wood, with other minor influences, such as residential heating oil. Differences in MLRs of OP^total DTT^ and OP^WS DTT^ suggested that BBOA contains more soluble species than the combined EC and BBOA. Cu likely had multiple sources, such as contributions from vehicles and wood burning, resulting in a lack of correlation with a specific source tracer.

### 3.4 Comparison of Two Winter Pollution Events in Fairbanks

To further assess factors influencing OP^DTT^ and OP^OH^, the two pollution events shown in Figure 1 were contrasted. The first event was in the coldest period of the study (average *T* = −27.2 °C) with the highest PM_2.5_ mass concentration (up to 89.0 μg/m^3^, 1 h-average). The other episode was toward the end of the study, when temperatures were significantly higher (average *T* = −5.3°C), although still with relatively high PM_2.5_ mass concentrations (31.0 μg/m^3^). High PM_2.5_ mass concentrations during both events were driven by strong temperature inversions, but PM during the events had different chemical characteristics (Figure 2). Figures 1 and 3 show that there was also a distinctly different relationship between the OP measurements.

In the first event, the sulfate mass fraction was high at 24%, while the OA (BBOA + HOA + POA2) contributed 61% (Figure 2b). BBOA and HOA accounted for 34% and 10% of the PM mass fraction, respectively. For species identified in the MLR models, concentrations of WS Fe, WS Cu, BBOA, and PAHs were approximately twice as high as the average levels observed throughout the entire study period, while Fe was similar (Tables S3 and S4). During this event, both OP_v_^total DTT^ and OP_v_^WS DTT^ values were at their highest level throughout the study period (Figure 1). These results are consistent with the MLR models, which suggest that BBOA was the primary contributor to OP_v_^DTT^ when considering ambient mass concentrations.

During the second pollution event, OA was again the major component of PM_2.5_, contributing 65% of the total PM_2.5_ mass, but HOA was the dominant OA component accounting for 30% of the total PM_2.5_ mass. On the other hand, the BBOA mass fraction dropped from 34% to 18%, and the sulfate mass fraction dropped from 24% to 17% (Figure 2c), both consistent with less residential heating emissions (biomass and fuel oil) in the warm vs cold events. The EC concentration was slightly higher in this event compared to the study average (0.60 vs 0.44 μg/m^3^). Water-soluble Fe was lower during this event, and the total Fe was comparable to the study mean value (Tables S3 and S5). OP_v_^OH^ was the highest observed throughout the study (Figure 1). The concurrent peak in OP_v_^OH^ and HOA supports the findings of the MLR models, which suggest that HOA was a significant contributor to the variability of OP_v_^OH^.

The overall results suggested that OP_v_^DTT^ is more responsive to residential heating emissions in Fairbanks. The peak of OP^DTT^ shown in the first pollution event was predominantly driven by a significant amount of wood (BBOA) and oil combustion (a sulfur source) from residential heating during the extremely low temperatures, with a lower fraction from vehicular emissions, whereas OP_v_^OH^ appears to be more responsive to vehicle emissions, which drove the OP^OH^ and HOA concurrent peak during the second pollution event. Note that mixtures of sources also contribute to the OP response; EC can come from both vehicles and wood burning. Biomass burning aerosols may also contribute to OP_v_^OH^ since WS Fe was predominately from wood burning based on the correlations (*r* = 0.91 for WS Fe – BrC). Furthermore, MLR results with the selection of different groups of species all suggest that BBOA and BrC, both associated with biomass burning, are substantial contributors to OP_v_^OH^ (see S25–S27).

### 3.5 Comparison of PM_2.5_ OP in Fairbanks with Atlanta and Los Angeles in Winter

A comparison of OP and PM_2.5_ mass concentrations between Fairbanks, Atlanta, and Los Angeles provides broad insights on sources and chemical species that drive OP, and how these OP assays characterize the relative air quality of these cities compared to using PM_2.5_ mass concentration. For consistency with Fairbanks, we compared data only during winter (cold) seasons.

These cities were chosen because they have data available for comparison and have contrasting emissions. Atlanta and Fairbanks data were from the analysis of filters at Georgia Tech. For Atlanta, we used the data from a previous study^38^ that was based on filters collected throughout 2017 at the Jefferson Street Site (representative of urban Atlanta with no strong nearby sources^83^). In that study, OP^OH^ was not determined, so archived filters were analyzed following the same method used for the Fairbanks samples. Underestimation of OP^OH^ levels due to the decay of certain OP^OH^-responsive species during the extended storage period of the filter samples is possible. For LA, we used PM_2.5_ mass, chemical composition (metals), and OP^OH^ and OP^DTT^ data recently reported by Shen et al. (2022).^73^ These data were from samples collected throughout LA and the surrounding region in February 2022 (27 samples). Both studies used the same measurement methods with a constant PM_2.5_ mass concentration of 10 μg/mL for OP^DTT^ and 25 μg/mL for OP^OH^ in the reaction vial, except for the OP^DTT^ measurement for Atlanta.

There are substantial contrasts in emissions and PM_2.5_ composition among the cities. Atlanta is influenced by biogenic SOA (terpenes in winter), vehicle emissions, and sulfate from large electrical generating units, although sulfate concentrations have been dropping.^84^ Biomass combustion from extensive prescribed burning in the region is also a significant contributor in winter, with estimates ranging from 18% to 50% of the measured PM_2.5_ mass.^85,86^ LA is dominated by vehicle emissions, with additional contributions from secondary sulfate and nitrate and marine aerosols.^73,87^ Fairbanks is dominated by residential heating emissions, including both biomass and fuel oil, and in contrast to Atlanta and LA, Fairbanks has reduced photochemical processing.

Despite low oxidant concentrations (i.e., O_3_) in Fairbanks, OP_v_^WS DTT^ was 77% of OP_v_^total DTT^ (discussed above) and so, on average, insoluble species contributed 23% to OP_v_^total DTT^. This insoluble fraction is lower than what has been found in Atlanta with higher oxidant concentrations (i.e., O_3_ of 21.8 ppb in Atlanta winter vs 7.8 ppb in Fairbanks winter), where 34% of the OP_v_^total DTT^ was due to insoluble species.^38^ We attribute this to the dominant contribution of BBOA (residential heating with wood) to OP_v_^total DTT^ in Fairbanks, which can contain oxygenated OA.^88^ The study average AMS-measured oxygen-to-carbon (O:C) ratio for Fairbanks OA was 0.39, at the upper end of the range reported for BBOA (0.1 to 0.4).^89^

**Figure 4:**
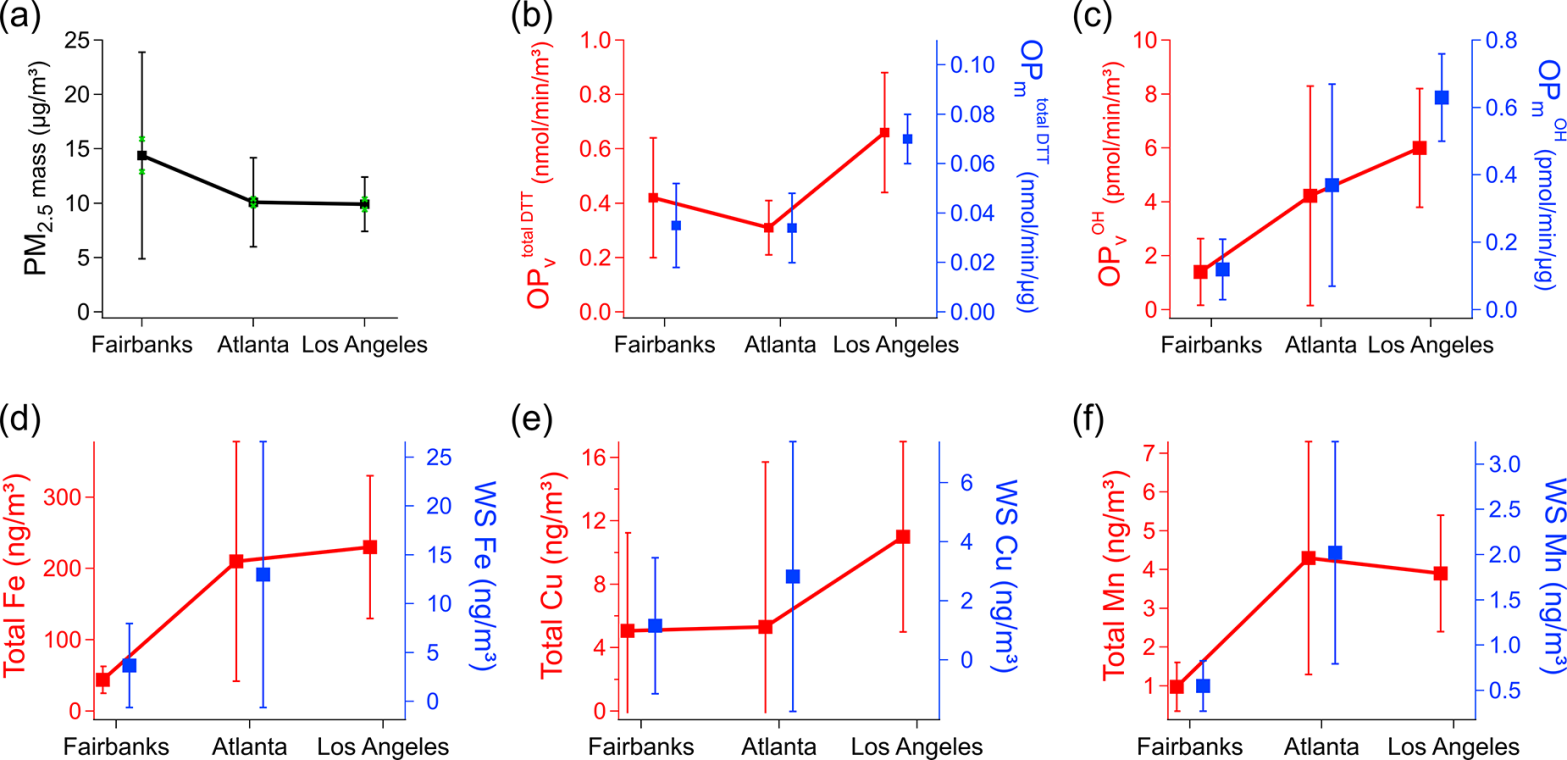
Mean wintertime PM_2.5_ (a) mass concentration, (b) OP^total DTT^, (c) OP^OH^, (d) Fe, (e) Cu, and (f) Mn of fine particulate matter collected in Fairbanks and previous studies in Atlanta^38^ and Los Angeles.^73^ The error bar represents the standard deviation of the mean. Both volume and mass normalized OP are shown. Total and water-soluble (WS) metals are plotted. There was no WS metal data available for Los Angeles. No speciated OA data were available for all three sites so are not included.

Figure 4 summarizes the comparisons of PM_2.5_ mass concentrations, OP^total DTT^, OP^OH^, and total and water-soluble metals (Fe, Cu, and Mn) between these cities (additional data given in Tables S6 and S7). PM_2.5_ mass concentration was somewhat similar amongst the three cities (Figure 4a), with Fairbanks having about 40 to 50% higher mean wintertime PM_2.5_ mass concentration and a much larger variation (14.4 ± 9.5 μg/m^3^) compared to Atlanta (10.1 ± 4.1 μg/m^3^) and Los Angeles (9.3 ± 2.5 μg/m^3^). For OP_v_^total DTT^, Atlanta had the lowest value (mean of 0.31 nmol/min/m^3^), while LA is higher by a factor of 2 (0.66 nmol/min/m^3^), and Fairbanks had a value between the two cities (0.42 nmol/min/m^3^). Additionally, Fairbanks and LA had a wider range in OP_v_^DTT^ than Atlanta (Fig. 4b). For OP_v_^OH^, the differences between these cities were more dramatic (Figure 4c). Atlanta had almost three times higher OP_v_^OH^ (4.23 pmol/min/m^3^) compared to Fairbanks (1.40 pmol/min/m^3^), while Los Angeles was 4.3 times higher (6.0 pmol/min/m^3^) than Fairbanks. These trends were the same for OP on a per mass basis (Figure 4b,c), meaning that the differences apply to both exposure (OP_v_) and the intrinsic health-related properties of PM_2.5_ (OP_m_).

As noted from the Fairbanks data, differences between the two OP methods were due to differences in the assay’s sensitivity to specific chemical species and hence sources. Both assays have been shown to be sensitive to certain metals and organic species, whereas past studies suggested that OP^DTT^ tends to have a broader sensitivity to OA than OP^OH^. Although no data for comparisons of speciated OA between these cities are available for the study periods, BBOA and HOA based on other studies in Atlanta and LA were lower than that in Fairbanks (Table S6), but these comparisons are more uncertain since they are not from the same sampling periods and locations as the OP and metals. A large difference between cities is the much lower concentrations of some metals, such as iron, in Fairbanks (see Figure 4).

We have shown that OP_v_^DTT^ is largely affected by Cu and biomass burning emissions. Past studies showed that OP^DTT^ in the Atlanta winter was largely linked to biomass burning (47%), followed by vehicles (12%).^90^ Thus, a somewhat higher OP^DTT^ value in Fairbanks may be due to higher contributions from incomplete combustion OA, which is dominated by emissions from wood heating, offsetting its somewhat lower Cu and EC concentrations from vehicles.

LA OP^DTT^ was significantly higher than Fairbanks or Atlanta, which could be driven by substantially higher Cu concentrations or a higher contribution from interactions between specific OA species and metals, (i.e., EC and Cu for OP_v_^total DTT^; see Supplemental Equations S29 to S32). Additionally, the contribution of SOA species might play a role in LA. It is noted that due to the low photochemically-driven processes in Fairbanks, the MLR analysis would not include a significant contribution from SOA, most importantly anthropogenic SOA, which includes highly oxidized and aromatic compounds,^91^ which could be one of the major contributors to OP in Atlanta and LA.

Atlanta shows an OP_v_^OH^ level three times as high as that of Fairbanks, while LA has an OP_v_^OH^ level nearly five times higher. Although the HOA in Atlanta and LA could be lower compared to Fairbanks, the elevated OP_v_^OH^ in these cities could mainly be attributed to significantly higher levels of Fe, and possible Fe interaction with HOA (e.g., see Supplemental Equations S29 to S32). Fe is a major contributor to OP_v_^OH^ levels in LA and Atlanta, whereas OA species are the main contributors in Fairbanks based on MLR analysis. This disparity follows from more differences in vehicle emissions in Atlanta and LA. The Fairbanks analysis showed OP^OH^ was more sensitive to vehicle emissions, and Shen et al. (2022)^73^ (the source of the LA OP data used here) found that 63% of OP_v_^OH^ and 42% of OP_v_^total DTT^ were from vehicle-related emissions in LA winter. The trends are also consistent with the expected differences in emissions. There were 8.0M automobiles, commercial vehicles, and motorcycles registered in the County of Los Angeles as of the year 2021;^92^ approximately 5M cars registered in Fulton County (the county comprising 90% of metropolitan Atlanta) in 2020,^93^ and 0.12M vehicles in Fairbanks-North Star Borough in 2022.^94^

Overall, among the three cities, OP^DTT^ and OP^OH^ showed different rankings compared to PM_2.5_ mass concentration. These differences hold for both volume- and mass-normalized OP. Based on average PM_2.5_ mass concentrations during the winter season, Fairbanks had the worst air quality compared to Atlanta and LA. However, an assay sensitive to a relatively broad panel of species, like OP^DTT^, suggests Fairbanks in winter was not substantially worse than Atlanta, where the higher OA emissions from residential heating using wood may be offset by the much lower traffic emissions in Fairbanks. However, the much higher traffic emissions in LA are the likely cause for its substantially higher OP^DTT^ and OP^OH^, where the difference was greatest for the assay notably sensitive to metals (OP^OH^) and primarily linked to vehicle emissions in Fairbanks. These results apply to both exposures (OP normalized by volume of air, OP_v_), and the intrinsic health-relevant properties of PM (OP normalized by particle mass, OP_m_). This study confirms that key components, including certain chemical forms of copper, iron, and aromatic-containing species, largely drive stable forms of OP (e.g., those determined with filter measurements), which is germane to the oxidative stress-related health effects of PM. These results suggest that specific acellular assays could be utilized to provide insights into exposures to certain emissions and their interactions when sources are not known^31^, such as in this case, OP^DTT^ for predominately incomplete combustion, and OP^OH^ for vehicle emissions, as well as biomass burning. Other assays may expand this to additional sources. These OP assays give a different view than PM_2.5_ mass concentration when contrasting air quality between these urban areas because of the differences in OP_m_, questioning the practice of relying solely on PM_2.5_ mass concentration to predict adverse health effects in all locations.

## Data Availability

Data is available on arcticdata.io: https://arcticdata.io/catalog/view/doi%3A10.18739%2FA23R0PV7J.

## Supporting Information Available

Additional experimental details, materials, and methods (Text S1–S5); results of multiple linear regression with the selection of different groups of species (Text S6–S7); statistical summary of different OP end points (Table S1); correlation between various PM2.5 components measured at the House site (Table S2); statistical summary of different PM2.5 chemical components (Tables S3–S5); mean and standard deviation of OP and various components of ambient PM2.5 in Fairbanks, Atlanta and Los Angeles in winter (Tables S6–S7); orthogonal regression and time-series of comparison between PM2.5 mass concentration at NCore site and the sum of various measured species at House site (Figure S1); comparison between total and water-soluble OPDTT (Figure S2); correlations between OP end points and PM2.5 components (Figure S3) (PDF)
