# Supplementary material for: Assessing the Oxidative Potential of Outdoor PM2.5 in Wintertime Fairbanks, Alaska
Source: ACS EST Air. 2024 Feb 10;1(3):175–87. doi: 10.1021/acsestair.3c00066 (PMC10928717; doi:10.1021/acsestair.3c00066)
Supplement: Supplementary file 1 — ea3c00066_si_001.pdf [file ea3c00066_si_001.pdf]

Assessing the Oxidative Potential of Outdoor $PM_{2.5}$ in Wintertime Fairbanks, Alaska

Yuhan Yang[1], Michael A. Battaglia [1, a], Magesh Kumaran Mohan[1], Ellis S. Robinson[2], Peter F. DeCarlo[2], Kasey C. Edwards[3], Ting Fang[3, b], Sukriti Kapur[3], Manabu Shiraiwa[3], Meeta Cesler-Maloney[4], William R. Simpson[4], James R. Campbell[4], Athanasios Nenes[1,5,6], Jingqiu Mao[4], Rodney J. Weber[1,]*

[1] School of Earth and Atmospheric Sciences, Georgia Institute of Technology, Atlanta, Georgia 30332, USA

[2] Department of Environmental Health & Engineering, Johns Hopkins University, Baltimore, Maryland, 21218 USA

[3] Department of Chemistry, University of California, Irvine, California, 92697 USA.

[4] Geophysical Institute and Department of Chemistry & Biochemistry, University of Alaska Fairbanks, Fairbanks, Alaska, 99775 USA

[5] Laboratory of Atmospheric Processes and their Impacts (LAPI), School of Architecture, Civil & Environmental Engineering, Ecole Polytechnique Fédérale de Lausanne, Lausanne, 1015 Switzerland.

[6] Center for Studies of Air Quality and Climate Change, Institute of Chemical Engineering Sciences, Foundation for Research and Technology Hellas, Patras,26504 Greece.

[a] Now at: U.S. Army DEVCOM CBC, Aberdeen Proving Ground, Maryland, 21010 USA

[b] Now at: Sustainable Energy and Environment Thrust, The Hong Kong University of Science and Technology (Guangzhou), Guangzhou, 511400 China

S.1 Acellular oxidative potential measurement

S.1.1 Water-soluble and total DTT measurement

The water-soluble DTT assay was conducted using a semi-automated system developed by Fang et al. (2015), ([1]) following the protocol described by Cho et al. (2005). ([2]) To perform the assay, the filtered PM extract (3.5 mL) was incubated with DTT solution (1 mM; 0.5 mL) and potassium phosphate buffer (1 mL; purified by passing thru Chelex 100 Resin column to remove possible binding polyvalent metal ions before adjusting pH to ~7.4) at 37 °C and shaken at a rotational frequency of 400 rpm using a ThermoMixer (Eppendorf North America, Inc., Hauppauge, NY, USA). At designated times (4, 13, 23, 31 and 41 min), a small aliquot (100 µL) of the mixture was withdrawn and mixed with trichloroacetic acid (TCA, 1% w/v; 1 mL) to quench the DTT reaction. Tris buffer (2 mL; mixed with ethylenediaminetetraacetate (EDTA) to remove possible metal contamination; pH ~8.9) and 5,5'-dithiobis-(2-nitrobenzoic acid) (DTNB, 0.2 mM; 0.5 mL) were added to the mixture with residual DTT to form 2-nitro-5-thiobenzoic acid (TNB), a colored product with light absorption at 412 nm. The final mixture was injected into a 0.1 m path-length liquid waveguide capillary cell (LWCC; World Precision Instruments, Inc., FL, USA), and the light absorption was recorded by an online spectrometer, which included a UV-Vis (200 to 800 nm) dual deuterium and tungsten halogen light source (DT-mini-2, Ocean Optics, Inc., Dunedin, FL, USA), and a multi-wavelength light detector (USB4000 Miniature Fiber Optic Spectrometer).

The quantification of $OP^{total\ DTT}$ was carried out using a semi-automated system developed by Gao et al. (2017), ([3]) following the same protocol as the $OP^{WS\ DTT}$ measurement with the exception that the insoluble PM components suspended in the extracts or attached to the filter surface could also participate in DTT oxidation by performing the assay with the filter in the reagents.

The DTT consumption rate was determined by the slope of the linear regression of DTT residual versus reaction time. A field blank and a positive control (9,10-phenanthraquinone) were analyzed with all sample batches. The PM OP measured by this DTT assay was blank-corrected and normalized by the air volume that passed through the extracted filter (volume-normalized, OP_v^{DTT}) or the PM mass loading on the extracted filter (mass-normalized, OP_m^{DTT}). The assay was performed at an approximate particle concentration of 10 µg/mL for the analysis of Fairbanks and LA samples, but not for Atlanta samples.

S.1.2 Hydroxyl radical generation in surrogate lung fluid

The OH assay was performed following the protocol described by Yu et al. (2021). ([4]) The PM extract was mixed with surrogate lung fluid (SLF) consisting of a mixture of 2.5 mM ascorbic acid (AA), 1.25 mM reduced glutathione (GSH), and uric acid (UA), which was dissolved in phosphate-buffered saline (PBS, PH ~7.4) along with potassium phosphate-buffered disodium terephthalate (TPT) (50 mM; pH ~7.4). TPT captures OH radicals generated in the reaction and forms 2-hydroxyterephthalic acid (2-OHTA), a fluorescent product that can be measured at an excitation/emission wavelength of 310/427 nm. The mixing solution was incubated at 37 °C and shaken at a rotational frequency of 400 rpm. At selected time intervals (30, 60, 90 and 120 min), aliquots of this reaction mixture (2 mL) were withdrawn and mixed with dimethyl sulfoxide (DMSO, 100 mM; 1 mL) to quench the reaction between ·OH and TPT. The final mixture was analyzed by a Shimadzu Spectro fluorophotometer (RF-5301PC). The excitation and emission slit widths were set at 5 nm and 10 nm, respectively. The concentration of 2-OHTA was determined by calibrating with 10 different concentrations (0–500 nM) of 2-OHTA standards, and the generation rate of ·OH was determined as the formation rate of 2-OHTA divided by a yield factor

of 0.35. ([5]) The PM OP measured by this OH assay was blank-corrected and normalized by the air volume that passed through the extracted filter (volume-normalized, OP_v^{OH}) or the PM mass loading on the extracted filter (mass-normalized, OP_m^{OH}). The assay was performed at an approximate particle concentration of 25 μg/mL for the analysis of the samples from all three cities.

S.2 Aerosol mass spectrometry measurements

A High-Resolution Time-of-Flight Aerosol Mass Spectrometer (HR-ToF-AMS, Aerodyne Research, Inc., USA) was used for online measurement of non-refractory sub-micron PM (NR-PM1) composition and mass concentrations. Positive matrix factorization (PMF) was applied to AMS organic aerosol mass spectra and a set of factors corresponding to specific sources or types of compounds were determined. The overall data set included mass concentrations of NH_4^+, NO_3^-, SO_4^{2-}, Cl^-, and OA; OA components from PMF; polycyclic aromatic hydrocarbon (PAH) concentrations; and oxygen-to-carbon (O:C) ratio for the OA. The OA components include factors from a three-factor PMF solution (described in more detail below): hydrocarbon-like OA (HOA), biomass-burning OA (BBOA), and another primary OA (POA2). The instrument was connected to a sampling line (built with 3/8 inch-diameter copper tubing) fitted with a PM_1 inlet positioned outside the house at 1.5 m above ground. The total sampling flow was 5.5 L/min. Prior to the AMS measurements, the aerosol was dried with a Nafion dryer (MD-070, Perma Pure LLC., USA) to reduce the relative humidity (RH) below 30%.

The three-factor PMF solution we present here was run on the entire AMS dataset, which included alternating outdoor and indoor sampling at equal time intervals. There was a series of indoor experiments conducted in the house to explore interactions between outdoor-infiltrating

and indoor-generated aerosols, but these periods were excluded from this PMF analysis. The HOA and BBOA factors identified in this PMF solution have mass spectra very similar to canonical examples from the literature. We performed a correlation analysis and found our HOA to have an average Pearson correlation coefficient of 0.97 with spectra from Elser et al ([6]) and Mohr et al ([7]). Similarly, our BBOA factor has an average Pearson correlation coefficient of 0.75 with Elser et al ([6]). This correlation coefficient is lower than the respective value for HOA compared to literature, but BBOA mass spectra are typically highly variable, and so in that context, the correlation coefficient is still high. We also see characteristic mass spectral fragments in our BBOA factor (e.g., $C_2H_4O_2^+$, a tracer fragment for levoglucosan) and expected diurnal pattern. The remaining mass was apportioned to a factor that we are calling "POA2", though we are not sure exactly what source(s) may contribute to it. We name it "POA2" because it does not resemble the mass spectrum of any canonical secondary OA or oxidized OA. Rather, it has strong contributions from C_xH_y fragments and high signal at m/z 55, both of which are characteristic of a variety of primary OA spectra (e.g., cooking, vehicles, and others).

We quantified total PAHs, per the method introduced by Dzepina et al ([8]). This approach uses unit-mass resolution fragmentation patterns established by Dzepina et al., which are built into the SQUIRREL (v.1.65C) AMS analysis software. We were unable to resolve our PAH signal into sub-classes per the methods of Herring et al. ([9]), as our mass spectra lacked the proper resolution at high (>200) m/z to differentiate between possible candidate ion fragments. High m/z is where most PAH fragments are located due to their resistance to fragmentation. We operated the instrument in V-mode using a mass range up to m/z 405.

O:C was calculated using the PIKA (v.1.25C) analysis software, using the “improved ambient” method introduced by Canagaratna et al ([10]).

S.3 Elemental Analysis

To measure the water-soluble elements, one (1-inch diameter, 5.06 cm^2) punch was extracted in 5 mL of DI water in a sterile polypropylene centrifuge tube via a 60-min ultrasonic treatment. The extracts were then filtered using a 0.45 µm PTFE syringe filter and the filtered extract was acid-preserved with concentrated nitric acid (70%) to a final concentration of 2% (v/v) to maintain the metals in solution. Metals in the filtrate are defined as water-soluble metals, which encompass all dissolved forms as well as any colloidal particles with a diameter of less than 0.45 µm. ([12])

One 1.5 cm^2 filter punch from the Hi-Vol quartz filter was acid-digested using aqua regia (HNO_3+3HCl) to quantify the total metal concentration. The filter was incubated in the acid at 99 °C while shaken at a rotational frequency of 400 rpm using a ThermoMixer for 24 h. The acid-digested sample was then diluted in DI water and filtered through a 0.45 µm PTFE syringe filter. ([12])

Both total and water-soluble elements were measured by inductively coupled plasma mass spectrometry (ICP-MS, Agilent 7500a series, Agilent Technologies, Inc., CA, USA) using EPA method 6020. ([13]) Calibrations were performed at the beginning of every measurement day using a dilution of commercially available standard stock solutions (1000 ppm in 2% HCl), and the data were blank-corrected. The concentrations of various elements including magnesium, aluminum, potassium, manganese, iron, copper, zinc and lead are included in the analysis. Other elements were measured but deemed less useful or were mostly below detection limits.

S.4 Organic carbon and elemental carbon and brown carbon analysis

To determine the organic carbon (OC) and elemental carbon (EC) content of fine particulate matter, a 1.5 cm^2 filter punch was analyzed with a benchtop Sunset OC/EC analyzer (Sunset Laboratory Inc. OR, USA). The measurement was carried out following the National Institute for Occupational Safety and Health (NIOSH) 5040 analysis protocol. ([14])

For the determination of brown carbon (BrC), a 1.5 cm^2 filter punch from the Hi-Vol filter was extracted in 15 mL DI or methanol by 60 min sonication. The light absorption of soluble BrC species was measured with a liquid-based spectrophotometric method in each of these solvent extracts that was filtered (0.22 μm pore size syringe filter) and then injected into a 2.5 m path LWCC, which was coupled with a broadband UV–Vis–NIR light source and a spectrometer. As a measure of BrC levels (i.e., chromophores), the light absorbance at 365 nm (average of 360-370 nm) wavelength was determined for each solvent (A_{365}) and the absorption coefficient was calculated by ([15]):

$$Abs_{365} = (A_{365} - A_{700})\frac{V_l}{V_a \cdot l}\ln(10)$$

where A_{700} is the average light absorbance at 695-705 nm, V_l the volume of water or methanol the filter was extracted into, which is 15 mL, V_a the volume of sample air that passed through the filter punch (based on overall flow rate and the ratio of punch area to overall filter collection area), and l the length of the waveguide, which is 2.5 m.

S.5 Description of stepwise regression

Stepwise regression involves a systematic process of adding and removing variables from the model, based on their statistical significance, until a final model with the best set of predictors is identified. Before regression analysis, extreme outliers (data points beyond an outer fence, which are lower than the lower quartile (Q1; 25th percentile) - 3*interquartile range (IQ; Q3-Q1) or higher than the upper quartile (Q3; 75th percentile) + 3*interquartile range; 2 data points in total) were removed. The inclusion or exclusion of variables is based on the Akaike information criterion (AIC) and Bayesian information criterion (BIC), which are widely accepted criteria for selecting the optimal model. There is no difference between the models selected by AIC and those selected by BIC.

The standardized MLR method involved rescaling the variables using a linear transformation such that all variables had a mean of 0 and variance of 1 (e.g., normalize each variable x by $(x - \bar{x})/\sigma$), allowing for a more effective comparison of their relative importance, where $\bar{x}$ is the mean and σ the standard deviation of that variable. The standardized coefficients were used to interpret the change in OP that would occur for a one-unit change in the predictor variable. The absolute values of these standardized regression coefficients represented the relative importance of the variables.

S.6 Multiple Linear Regression results with the selection of different groups of species

The unstandardized and standardized regression results for the selection of different groups of species selected from those that were correlated are shown below:

Unstandardized Regression with different independent variables that were correlated:

The first equation in each group in italics, is the regression included in the main text of the paper.

$$OP_v^{total\ DTT} = 0.309\ EC + 0.036\ BBOA + 13.84\ Cu + 0.078 \qquad r^2 = 0.81,\ MSE = 0.010 \qquad (S1)$$

$$\mathrm{OP_v^{total\ DTT} = 10.5\ PAH + 17.97\ Cu + 0.090} \qquad \mathrm{r^2 = 0.82,\ MSE = 0.008} \qquad \mathrm{(S2)}$$

$$\mathrm{OP_v^{total\ DTT} = 0.439\ EC + 0.041\ MS\ BrC + 12.52\ Cu + 0.018} \qquad \mathrm{r^2 = 0.81,\ MSE = 0.013} \qquad \mathrm{(S3)}$$

$$\mathrm{OP_v^{total\ DTT} = 0.482\ EC + 0.084\ WS\ BrC + 12.25\ Cu + 0.014} \qquad \mathrm{r^2 = 0.80,\ MSE = 0.014} \qquad \mathrm{(S4)}$$

$$OP_v^{WS\ DTT} = 0.058\ BBOA + 11.68\ Cu + 0.043 \qquad r^2 = 0.88,\ MSE = 0.005 \qquad (S5)$$

$$\mathrm{OP_v^{WS\ DTT} = 0.148\ WS\ BrC + 8.83\ Cu + 0.024} \qquad \mathrm{r^2 = 0.82,\ MSE = 0.008} \qquad \mathrm{(S6)}$$

$$\mathrm{OP_v^{WS\ DTT} = 0.066\ MS\ BrC + 9.36\ Cu + 0.026} \qquad \mathrm{r^2 = 0.82,\ MSE = 0.008} \qquad \mathrm{(S7)}$$

$$\mathrm{OP_v^{WS\ DTT} = 10.36\ PAH + 15.03\ Cu + 0.019} \qquad \mathrm{r^2 = 0.77,\ MSE = 0.010} \qquad \mathrm{(S8)}$$

$$\mathrm{OP_v^{WS\ DTT} = 0.085\ POA2 + 15.46\ Cu + 0.021} \qquad \mathrm{r^2 = 0.72,\ MSE = 0.021} \qquad \mathrm{(S9)}$$

$$OP_v^{OH} = 0.332\ HOA + 89.34\ WS\ Fe + 13.84\ Fe - 0.306 \qquad r^2 = 0.82,\ MSE = 0.288 \qquad (S10)$$

$$\mathrm{OP_v^{OH} = 0.290\ HOA + 0.158\ BBOA + 13.69\ Fe - 0.502}$$

$$r^2 = 0.82, MSE = 0.294 \qquad (S11)$$

$$OP_v^{OH} = 0.300\ HOA + 0.396\ WS\ BrC + 13.89\ Fe - 0.603$$

$$r^2 = 0.82, MSE = 0.298 \qquad (S12)$$

$$OP_v^{OH} = 0.297\ HOA + 0.173\ MS\ BrC + 14.08\ Fe - 0.582$$

$$r^2 = 0.81, MSE = 0.313 \qquad (S13)$$

$$OP_v^{OH} = 0.202\ HOA + 0.278\ POA2 + 20.04\ Fe - 0.714$$

$$r^2 = 0.81, MSE = 0.320 \qquad (S14)$$

Standardized Regression:

$$OP_v^{total\ DTT} = 0.308\ EC + 0.403\ BBOA + 0.273\ Cu - 0.047$$

$$r^2 = 0.81, MSE = 0.146 \qquad (S15)$$

$$OP_v^{total\ DTT} = 0.584\ PAH + 0.354\ Cu - 0.068 \qquad r^2 = 0.82, MSE = 0.133 \qquad (S16)$$

$$OP_v^{total\ DTT} = 0.438\ EC + 0.397\ MS\ BrC + 0.247\ Cu \qquad r^2 = 0.81, MSE = 0.206 \qquad (S17)$$

$$OP_v^{total\ DTT} = 0.481\ EC + 0.362\ WS\ BrC + 0.241\ Cu \qquad r^2 = 0.80, MSE = 0.214 \qquad (S18)$$

$$OP_v^{WS\ DTT} = 0.805\ BBOA + 0.284\ Cu - 0.015 \qquad r^2 = 0.88, MSE = 0.128 \qquad (S19)$$

$$OP_v^{WS\ DTT} = 0.793\ WS\ BrC + 0.215\ Cu \qquad r^2 = 0.82, MSE = 0.185 \qquad (S20)$$

$OP_v^{WS\ DTT} = 0.782\ MS\ BrC + 0.228\ Cu$ $r^2 = 0.82, MSE = 0.194$ (S21)

$OP_v^{WS\ DTT} = 0.712\ PAH + 0.366\ Cu – 0.014$ $r^2 = 0.77, MSE = 0.240$ (S22)

$OP_v^{WS\ DTT} = 0.673\ POA2 + 0.376\ Cu – 0.014$ $r^2 = 0.72, MSE = 0.294$ (S23)

$OP_v^{OH} = 0.523\ HOA + 0.290\ WS\ Fe + 0.196\ Fe – 0.071$

$r^2 = 0.82, MSE = 0.161$ (S24)

$OP_v^{OH} = 0.457\ HOA + 0.366\ BBOA + 0.194\ Fe – 0.081$

$r^2 = 0.82, MSE = 0.164$ (S25)

$OP_v^{OH} = 0.473\ HOA + 0.323\ WS\ BrC + 0.196\ Fe – 0.075$

$r^2 = 0.82, MSE = 0.166$ (S26)

$OP_v^{OH} = 0.467\ HOA + 0.315\ MS\ BrC + 0.199\ Fe – 0.073$

$r^2 = 0.81, MSE = 0.174$ (S27)

$OP_v^{OH} = 0.318\ HOA + 0.334\ POA2 + 0.284\ Fe – 0.077$

$r^2 = 0.81, MSE = 0.179$ (S28)

The selection of different groups of species resulted in a consistent pattern of regression results, exhibiting similar coefficients, intercept, and overall model performance, as evidenced by

comparable values of r^2 and MSE. This consistency implies that the choice of species grouping did not significantly impact the models' predictive ability and explanatory power in most cases.

An exception was observed in the regression analysis of $OP_v^{total\ DTT}$, where an alternative combination of species, namely EC and BBOA or BrC, proved to be as effective as including PAHs. It is important to note that this observation is in line with the known characteristics of PAHs as hydrophobic organic compounds predominantly emitted from combustion processes such as biomass burning and fossil fuel combustion. Therefore, $OP_v^{total\ DTT}$ could be considered as a measure reflecting the combined impact of residential heating and vehicular emissions in Fairbanks.

S.7 Additional notes about multivariate linear regression

To explore the impact of synergistic and antagonistic interactions among various PM species on oxidative potential, interaction terms were included for fitting the MLR models. In the MLR model for $OP_v^{WS\ DTT}$, no interaction terms were found to be significant based on the stepwise regression analysis. Whereas, for $OP_v^{total\ DTT}$ and OP_v^{OH}, significant interaction term(s) were identified. Selected results are presented below (Eq S29-32):

Unstandardized Regression with interaction terms:

$OP_v^{total\ DTT} = 0.070\ \text{MS BrC} + 56.06\ \text{EC*Cu} - 2.88\ \text{MS BrC*Cu} + 0.138$

$r^2 = 0.84$, MSE = 0.011 (S29)

$OP_v^{OH} = 0.197\ \text{BBOA} + 4.52\ \text{HOA*Fe} - 0.018$ $r^2 = 0.86$, MSE = 0.228 (S30)

Standardized Regression with interaction terms:

$$OP_v^{total\ DTT} = 0.404\ EC + 0.385\ MS\ BrC + 0.197\ Cu + 0.209\ EC{*}Cu - 0.081$$

$$r^2 = 0.84,\ MSE = 0.179 \qquad (S31)$$

$$OP_v^{OH} = 0.442\ BBOA + 0.324\ Fe + 0.297\ HOA{*}Fe - 0.260$$

$$r^2 = 0.87,\ MSE = 0.124 \qquad (S32)$$

Considering the intercept, r^2 and MSE of the regression models, the inclusion of interaction terms had limited improvement in model fitting performance, except for OP_v^{OH}, which may indicate a synergistic effect of HOA and Fe on hydroxyl radical production rate. Therefore, the inclusion of interaction terms was not pursued. Additionally, due to the limited number of data points (~40), introducing higher-order terms or employing splines for nonlinear regression may lead to overfitting issues. Thus, MLR models were constructed without including any higher-order or nonlinear terms, assuming the effects of each predictor were additive. However, it is worth noting that this additive assumption may not always hold true in all cases.

Table S1. Statistical summary of different OP endpoints of $PM_{2.5}$ measured in Fairbanks (N = 40). Averaging time in all cases is 24 hours.

		Volume-normalized OP			Mass-normalized OP		
	$PM_{2.5}$ (µg/m³)	$OP_v^{total\ DTT}$ (nmol/min/m³)	$OP_v^{WS\ DTT}$ (nmol/min/m³)	OP_v^{OH} (pmol/min/m³)	$OP_m^{total\ DTT}$ (nmol/min/µg)	$OP_m^{WS\ DTT}$ (nmol/min/µg)	OP_m^{OH} (pmol/min/µg)
mean	14.42	0.42	0.33	1.40	0.035	0.026	0.119
std	9.48	0.22	0.21	1.24	0.017	0.013	0.085
min	2.16	0.09	0.05	LOD	0.013	0.008	LOD
25%	6.62	0.23	0.13	0.37	0.023	0.018	0.073
50%	12.28	0.43	0.32	1.10	0.034	0.022	0.099
75%	20.34	0.53	0.42	2.12	0.039	0.034	0.137
max	40.25	1.01	0.99	5.98	0.092	0.060	0.470

Table S2. Correlation between various $PM_{2.5}$ components measured at the House site in Fairbanks.

	Gas phase				Particle phase																
	SO_2[a]	CO[b]	NO[c]	NO_2[c]	$PM_{2.5}$ mass	SO_4	OC	EC	PAH	BBOA	HOA	POA2	NO_3	NH_4	WS BrC	MS BrC	Mn	Fe	Cu	Zn	Pb
$PM_{2.5}$ mass	0.85	0.83	0.86	0.86	1.00																
SO_4	0.90	0.68	0.73	0.82	0.75	1.00															
OC	0.85	0.66	0.69	0.81	0.89	0.79	1.00														
EC	0.55	0.77	0.84	0.71	0.82	0.38	0.71	1.00													
PAH	0.89	0.64	0.72	0.81	0.89	0.84	0.88	0.73	1.00												
BBOA	0.90	0.60	0.62	0.82	0.90	0.90	0.96	0.58	0.86	1.00											
HOA	0.40	0.88	0.86	0.60	0.75	0.49	0.58	0.85	0.53	0.46	1.00										
POA2	0.81	0.72	0.76	0.80	0.92	0.81	0.86	0.75	0.89	0.83	0.66	1.00									
NO_3	0.55	0.70	0.71	0.69	0.80	0.64	0.62	0.69	0.65	0.56	0.71	0.80	1.00								
NH_4	0.84	0.78	0.81	0.86	0.96	0.95	0.84	0.67	0.86	0.85	0.65	0.89	0.84	1.00							
WS BrC	0.85	0.53	0.57	0.79	0.82	0.76	0.98	0.62	0.85	0.95	0.42	0.79	0.53	0.78	1.00						
MS BrC	0.87	0.57	0.60	0.81	0.86	0.77	0.98	0.66	0.89	0.95	0.44	0.82	0.57	0.82	0.99	1.00					
WS Mn	0.79	0.66	0.72	0.78	0.86	0.75	0.85	0.69	0.84	0.84	0.56	0.83	0.70	0.85	0.82	0.83	0.71	0.71	0.27	0.06	0.33
WS Fe	0.87	0.47	0.54	0.70	0.77	0.75	0.88	0.53	0.83	0.90	0.28	0.72	0.44	0.73	0.91	0.91	0.43	0.58	0.28	0.02	0.17
WS Cu	0.70	0.45	0.51	0.58	0.65	0.59	0.79	0.61	0.74	0.72	0.36	0.68	0.47	0.61	0.80	0.81	0.43	0.58	0.67	-0.04	0.47

WS Zn	**0.50**	**0.51**	**0.54**	**0.58**	**0.56**	**0.47**	**0.60**	**0.47**	**0.59**	**0.61**	**0.45**	**0.66**	**0.60**	**0.61**	**0.58**	**0.56**	**0.55**	**0.42**	**0.5**	0.19	**0.58**
WS Pb	0.10	0.06	0.07	0.18	0.17	0.12	0.20	0.10	0.22	0.15	0.05	0.33	**0.42**	0.23	**0.21**	**0.20**	0.23	-0.02	0.62	0.08	**0.88**
Al	0.12	-0.02	-0.02	0.12	0.31	0.32	0.51	0.13	0.29	0.51	0.15	0.38	0.09	0.26	0.50	0.46	0.12	0.28	**0.28**	0.18	0.09
K	**0.62**	**0.63**	**0.62**	**0.65**	**0.62**	**0.63**	**0.69**	**0.54**	**0.69**	**0.66**	**0.56**	**0.57**	0.40	**0.63**	**0.66**	**0.65**	**0.45**	**0.59**	0.05	0.13	-0.04
Mn	**0.38**	**0.68**	**0.68**	**0.53**	**0.69**	**0.40**	**0.55**	**0.66**	**0.46**	**0.48**	**0.77**	**0.51**	**0.69**	**0.61**	**0.46**	**0.47**	**1.00**				
Fe	**0.58**	**0.86**	**0.76**	**0.59**	**0.81**	**0.58**	**0.75**	**0.80**	**0.69**	**0.66**	**0.77**	**0.69**	**0.61**	**0.72**	**0.65**	**0.69**	**0.67**	**1.00**			
Cu	0.25	0.12	0.11	0.18	0.20	0.07	0.33	**0.26**	0.17	0.21	0.08	0.22	0.10	0.09	**0.34**	**0.31**	0.20	0.17	**1.00**		
Zn	0.03	0.01	-0.11	0.17	0.07	-0.04	0.09	0.11	-0.02	0.08	-0.06	0.04	0.01	0.02	0.13	0.09	0.04	0.02	0.17	**1.00**	
Pb	-0.02	0.08	0.11	0.21	0.20	-0.04	0.17	**0.24**	0.15	0.05	0.09	0.29	0.37	0.14	0.17	0.18	0.31	0.06	**0.64**	0.15	**1.00**

a. Measurement made at 3m above the valley floor at the CTC site using Thermo Scientific 43C.

b. Measurement made at the HOUSE site using Picarro G2401.

c. Measurement made at the NCore site by Alaska Department of Environmental Conservation (ADEC).

Note: p-value < 0.01 are bold. Correlations not statistically significant (p-value > 0.05) are in grey. Pearson's r > 0.8 are in red.

Table S3. Statistical summary of different $PM_{2.5}$ chemical components measured in Fairbanks (N = 40).

	EC (μg/m³)	PAH (ng/m³)	BBOA (μg/m³)	HOA (μg/m³)	POA2 (μg/m³)	WS Mn (ng/m³)	WS Fe (ng/m³)	WS Cu (ng/m³)	WS Zn (ng/m³)	Cu (ng/m³)	Fe (ng/m³)
mean	0.437	22.4	3.85	2.19	2.68	0.549	3.66	1.16	3.06	5.06	44.1
std	0.250	13.8	2.82	2.09	1.59	0.283	4.30	1.01	2.26	6.17	18.9
min	0.154	1.94	0.153	0.154	0.402	0.158	LOD	LOD	LOD	LOD	16.2
25%	0.268	12.4	1.63	0.782	1.48	0.306	0.540	0.307	1.34	1.10	25.7
50%	0.334	18.7	3.67	1.60	2.52	0.470	1.30	0.828	2.42	3.02	42.2
75%	0.526	30.5	5.17	3.00	3.55	0.763	5.30	1.60	3.97	6.19	56.1
max	1.234	57.1	12.13	10.3	6.22	1.06	15.6	3.71	8.66	21.0	93.9

Table S4. Statistical summary of different $PM_{2.5}$ components and OP endpoints measured during pollution Event 1 (1/29/2022-2/4/2022, N = 6).

	$OP_v^{total\ DTT}$ (nmol/min/m³)	$OP_v^{WS\ DTT}$ (nmol/min/m³)	OP_v^{OH} (pmol/min/m³)	EC (µg/m³)	PAH (ng/m³)	BBOA (µg/m³)	HOA (µg/m³)	POA2 (µg/m³)	WS Mn (ng/m³)	WS Fe (ng/m³)	WS Cu (ng/m³)	Cu (ng/m³)	Fe (ng/m³)
mean	0.644	0.624	2.50	0.479	39.3	8.72	2.50	4.36	0.866	10.1	2.35	8.08	55.7
std	0.234	0.230	0.63	0.141	11.4	2.52	1.33	1.70	0.226	5.13	1.24	7.06	14.1
min	0.437	0.365	1.63	0.265	17.1	4.54	0.58	1.48	0.430	4.64	0.48	2.27	29.1
50%	0.571	0.635	2.41	0.498	42.5	8.69	2.52	4.59	0.949	11.6	2.38	6.25	60.6
max	1.007	0.989	3.55	0.680	48.7	12.1	4.08	5.99	1.05	15.6	3.71	21.0	66.9

Table S5. Statistical summary of different $PM_{2.5}$ components and OP endpoints measured during pollution Event 2 (2/22/2022-2/26/2022, N = 4).

	$OP_v^{total\ DTT}$ (nmol/min/m^3)	$OP_v^{WS\ DTT}$ (nmol/min/m^3)	OP_v^{OH} (pmol/min/m^3)	EC (µg/m^3)	PAH (ng/m^3)	BBOA (µg/m^3)	HOA (µg/m^3)	POA2 (µg/m^3)	WS Mn (ng/m^3)	WS Fe (ng/m^3)	WS Cu (ng/m^3)	Cu (ng/m^3)	Fe (ng/m^3)
mean	0.374	0.228	2.88	0.597	18.9	3.32	5.68	3.23	0.474	0.929	0.624	2.85	55.6
std	0.170	0.152	2.42	0.365	14.4	2.24	4.39	2.16	0.334	0.959	0.584	1.32	36.0
min	0.186	0.098	0.644	0.277	2.56	0.725	1.60	1.14	0.178	0.137	0.134	1.32	36.0
50%	0.365	0.208	2.44	0.575	18.0	3.47	5.40	2.79	0.473	0.755	0.517	3.22	52.1
max	0.580	0.399	5.98	0.960	37.1	5.60	10.3	6.22	0.770	2.07	1.33	4.01	93.9

Table S6. Mean and standard deviation of volume-normalized OP and various components of ambient $PM_{2.5}$ in Fairbanks (Jan-Feb), Atlanta and Los Angeles in winter.

	Fairbanks	Atlanta[a]	Los Angeles[b]
$PM_{2.5}$ mass, µg/m^3	14.4 ± 9.5	10.1 ± 4.1	9.3 ± 2.5
$OP_V^{WS\ DTT}$, nmol/min/m^3	0.33 ± 0.22	0.20 ± 0.07	
$OP_V^{total\ DTT}$, nmol/min/m^3	0.42 ± 0.22	0.31 ± 0.10	0.66 ± 0.22
OP_V^{OH}, pmol/min/m^3	1.40 ± 1.24	4.23 ± 4.07[c]	6.0 ± 2.2
EC, µg/m^3	0.44 ± 0.25	0.54 ± 0.38	0.50 ± 0.18
OC, µg/m^3	4.26 ± 2.41	3.00 ± 1.72	4.7 ± 0.3[d]
BBOA, µg/m^3	3.85 ± 2.82	0.72 ± 0.72[e]	< 0.8 (summer)[f]
HOA, µg/m^3	2.19 ± 2.09	0.51 ± 0.59[e]	1.2 (summer)[f]
Total Cu, ng/m^3	5.06 ± 6.17	5.32 ± 10.40[g]	11 ± 6
Total Fe, ng/m^3	44.1 ± 18.9	210 ± 168	230 ± 100
Total Mn, ng/m^3	0.98 ± 0.63	4.3 ± 3.0	3.9 ± 1.5
WS Cu, ng/m^3	1.16 ± 2.30	2.83 ± 4.56[g]	
WS Fe, ng/m^3	3.66 ± 4.30	13.0 ± 13.6	
WS Mn, ng/m^3	0.55 ± 0.28	2.02 ± 1.23	

a. Unless stated separately, ambient $PM_{2.5}$ data in Atlanta is obtained from Gao et al. (2020). ([16])

b. Unless stated separately, ambient $PM_{2.5}$ data in Los Angeles is obtained from Shen et al. (2022). ([17])

c. OP^{OH} analysis on selected filters collected in Atlanta in 2017 (N = 24, same sample sets used in Gao et al. (2020) ([16]))

d. Altuwayjiri et al. (2021) ([18])

e. Joo et al. (2021) ([19])

f. Summer data in Riverside, CA, Docherty et al. (2011) ([20])

g. Whole year data, Ye et al. (2018) ([21])

Table S7. Mean and standard deviation of mass-normalized OP and various components of ambient $PM_{2.5}$ in Fairbanks (Jan-Feb), Atlanta and Los Angeles in winter.

	Fairbanks	Atlanta[a]	Los Angeles[b]
$OP_m^{WS\ DTT}$, nmol/min/μg	0.025 ± 0.012	0.023 ± 0.009	
$OP_m^{total\ DTT}$, nmol/min/μg	0.035 ± 0.017	0.034 ± 0.014	0.070 ± 0.010
OP_m^{OH}, pmol/min/μg	0.12 ± 0.09	0.37 ± 0.30 (Nov-Dec)[c]	0.63 ± 0.13
EC, ng/μg	36 ± 17	51.3 ± 21.1	52 ± 12
OC, μg/μg	0.33 ± 0.10	0.29 ± 0.08	
Total Cu, ng/μg	0.40 ± 0.87	0.5 ± 1.0 (whole year)[d]	1.0 ± 0.4
Total Fe, ng/μg	4.10 ± 2.80	20.8 ± 15.0	23 ± 6
Total Mn, ng/μg	0.09 ± 0.05	0.44 ± 0.32	0.39 ± 0.09
WS Cu, ng/μg	0.08 ± 0.27	0.3 ± 0.4 (whole year)[d]	
WS Fe, ng/μg	0.20 ± 0.16	1.23 ± 0.93	
WS Mn, ng/μg	0.04 ± 0.02	0.19 ± 0.11	

a. Unless stated separately, ambient $PM_{2.5}$ data in Atlanta is obtained from Gao et al. (2020). (Gao, Mulholland, Russell and Weber [16])

b. Unless stated separately, ambient $PM_{2.5}$ data in Los Angeles is obtained from Shen et al. (2022). ([17])

c. OP^{OH} analysis on selected filters collected in Atlanta in 2017 (N = 24, same sample sets used in Gao et al. (2020) ([16])).

d. Estimated from Ye et al. (2018). ([21])

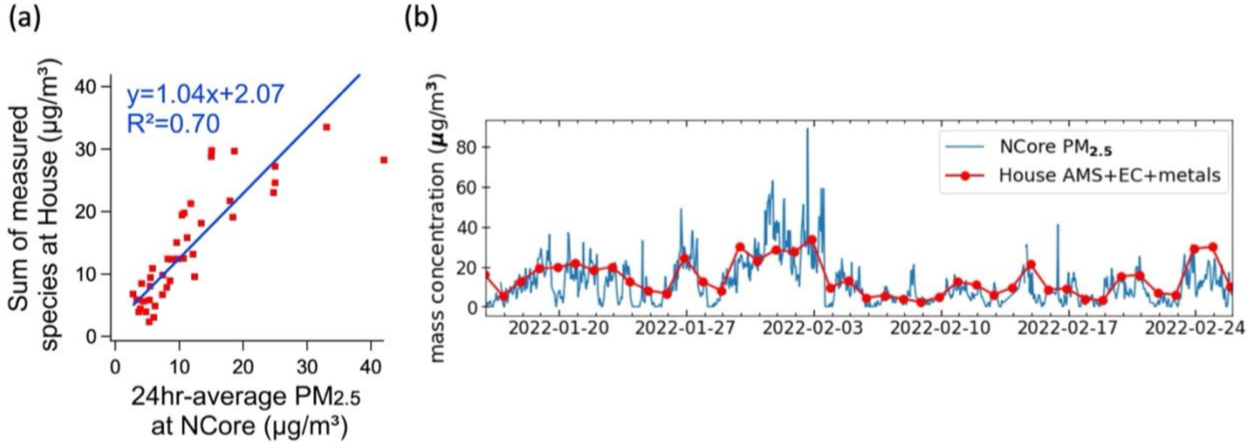

Figure S1. (a) Orthogonal regression and (b) time-series of comparison between $PM_{2.5}$ mass concentration at NCore site and the sum of various measured species (AMS Cl^-, NO_3^-, SO_4^{2-}, NH_4^+, OA, and EC and metals from the filters) at House site.

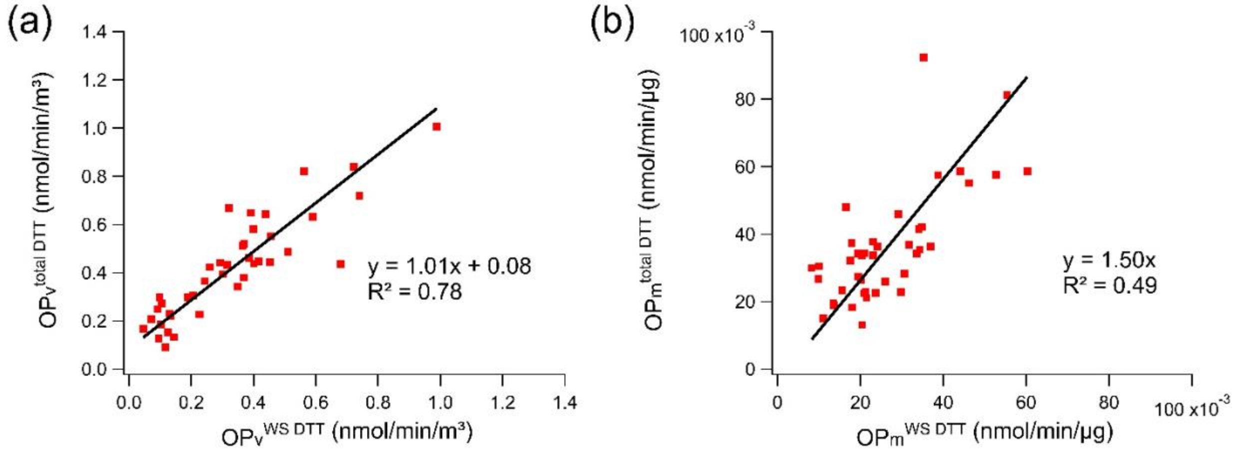

Figure S2. Comparison between total and water-soluble (a) volume-normalized and (b) mass-normalized OP^{DTT}.

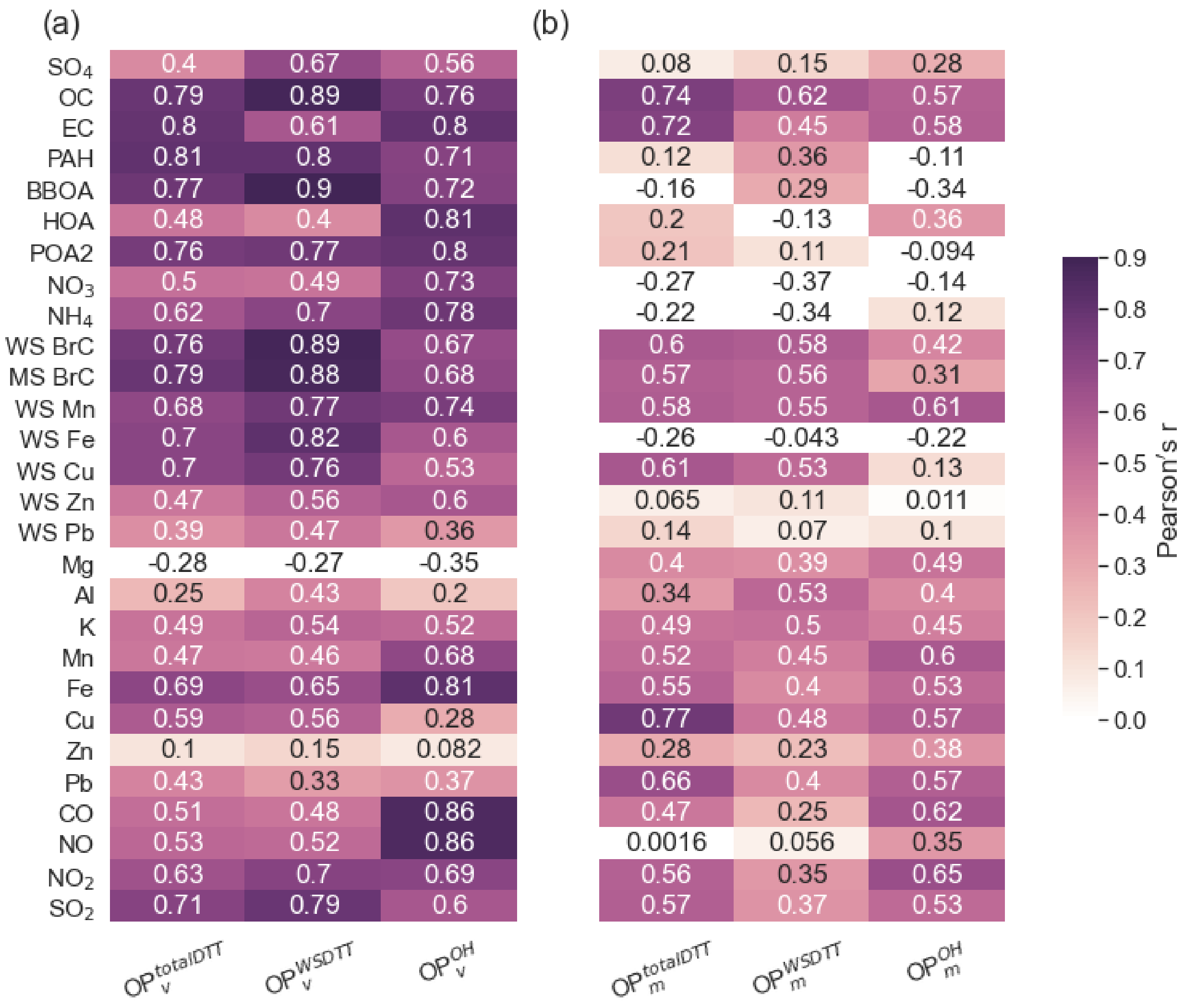

Figure S3. Correlations between (a) volume-normalized, (b) mass-normalized OP endpoints and PM components measured in Fairbanks.

REFERENCES

(1) Fang, T.; Verma, V.; Guo, H.; King, L.; Edgerton, E.; Weber, R. A semi-automated system for quantifying the oxidative potential of ambient particles in aqueous extracts using the dithiothreitol (DTT) assay: results from the Southeastern Center for Air Pollution and Epidemiology (SCAPE). Atmospheric Measurement Techniques 2015, 8 (1), 471-482.

(2) Cho, A. K.; Sioutas, C.; Miguel, A. H.; Kumagai, Y.; Schmitz, D. A.; Singh, M.; Eiguren-Fernandez, A.; Froines, J. R. Redox activity of airborne particulate matter at different sites in the Los Angeles Basin. Environmental research 2005, 99 (1), 40-47.

(3) Gao, D.; Fang, T.; Verma, V.; Zeng, L.; Weber, R. J. A method for measuring total aerosol oxidative potential (OP) with the dithiothreitol (DTT) assay and comparisons between an urban and roadside site of water-soluble and total OP. Atmos. Meas. Tech. 2017, 10 (8), 2821-2835. DOI: 10.5194/amt-10-2821-2017.

(4) Yu, H.; Puthussery, J. V.; Wang, Y.; Verma, V. Spatiotemporal variability in the oxidative potential of ambient fine particulate matter in the Midwestern United States. Atmospheric Chemistry and Physics 2021, 21 (21), 16363-16386.

(5) Son, Y.; Mishin, V.; Welsh, W.; Lu, S.-E.; Laskin, J. D.; Kipen, H.; Meng, Q. A novel high-throughput approach to measure hydroxyl radicals induced by airborne particulate matter. International journal of environmental research and public health 2015, 12 (11), 13678-13695.

(6) Elser, M.; Huang, R.-J.; Wolf, R.; Slowik, J. G.; Wang, Q.; Canonaco, F.; Li, G.; Bozzetti, C.; Daellenbach, K. R.; Huang, Y. New insights into PM 2.5 chemical composition and sources in two major cities in China during extreme haze events using aerosol mass spectrometry. Atmospheric Chemistry and Physics 2016, 16 (5), 3207-3225.

(7) Mohr, C.; DeCarlo, P.; Heringa, M.; Chirico, R.; Slowik, J.; Richter, R.; Reche, C.; Alastuey, A.; Querol, X.; Seco, R. Identification and quantification of organic aerosol from cooking and other sources in Barcelona using aerosol mass spectrometer data. Atmospheric Chemistry and Physics 2012, 12 (4), 1649-1665.

(8) Dzepina, K.; Arey, J.; Marr, L. C.; Worsnop, D. R.; Salcedo, D.; Zhang, Q.; Onasch, T. B.; Molina, L. T.; Molina, M. J.; Jimenez, J. L. Detection of particle-phase polycyclic aromatic hydrocarbons in Mexico City using an aerosol mass spectrometer. International Journal of Mass Spectrometry 2007, 263 (2-3), 152-170.

(9) Herring, C. L.; Faiola, C. L.; Massoli, P.; Sueper, D.; Erickson, M. H.; McDonald, J. D.; Simpson, C. D.; Yost, M. G.; Jobson, B. T.; VanReken, T. M. New methodology for quantifying polycyclic aromatic hydrocarbons (PAHs) using high-resolution aerosol mass spectrometry. Aerosol Science and Technology 2015, 49 (11), 1131-1148.

(10) Canagaratna, M.; Jimenez, J.; Kroll, J.; Chen, Q.; Kessler, S.; Massoli, P.; Hildebrandt Ruiz, L.; Fortner, E.; Williams, L.; Wilson, K. Elemental ratio measurements of organic compounds using aerosol mass spectrometry: characterization, improved calibration, and implications. Atmospheric Chemistry and Physics 2015, 15 (1), 253-272.

(11) Ulbrich, I.; Canagaratna, M.; Zhang, Q.; Worsnop, D.; Jimenez, J. Interpretation of organic components from Positive Matrix Factorization of aerosol mass spectrometric data. Atmospheric Chemistry and Physics 2009, 9 (9), 2891-2918.

(12) Yang, Y.; Gao, D.; Weber, R. J. A method for liquid spectrophotometric measurement of total and water-soluble iron and copper in ambient aerosols. Atmos. Meas. Tech. 2021, 14 (6), 4707-4719. DOI: 10.5194/amt-14-4707-2021.

(13) EPA, U. S. "Method 6020B (SW-846): Inductively Coupled Plasma-Mass Spectrometry," Revision 2. Washington, DC. 2014.

(14) Birch, M. E.; Cary, R. A. Elemental carbon-based method for monitoring occupational exposures to particulate diesel exhaust. Aerosol Science and Technology 1996, 25 (3), 221-241.

(15) Hecobian, A.; Zhang, X.; Zheng, M.; Frank, N.; Edgerton, E. S.; Weber, R. J. Water-Soluble Organic Aerosol material and the light-absorption characteristics of aqueous extracts measured over the Southeastern United States. Atmospheric Chemistry and Physics 2010, 10 (13), 5965-5977.

(16) Gao, D.; Mulholland, J. A.; Russell, A. G.; Weber, R. J. Characterization of water-insoluble oxidative potential of PM2. 5 using the dithiothreitol assay. Atmospheric Environment 2020, 224, 117327.

(17) Shen, J.; Taghvaee, S.; La, C.; Oroumiyeh, F.; Liu, J.; Jerrett, M.; Weichenthal, S.; Del Rosario, I.; Shafer, M. M.; Ritz, B.; et al. Aerosol Oxidative Potential in the Greater Los Angeles Area: Source Apportionment and Associations with Socioeconomic Position. Environmental Science & Technology 2022, 56 (24), 17795-17804. DOI: 10.1021/acs.est.2c02788.

(18) Altuwayjiri, A.; Pirhadi, M.; Taghvaee, S.; Sioutas, C. Long-term trends in the contribution of PM 2.5 sources to organic carbon (OC) in the Los Angeles basin and the effect of PM emission regulations. Faraday Discussions 2021, 226, 74-99.

(19) Joo, T.; Chen, Y.; Xu, W.; Croteau, P.; Canagaratna, M. R.; Gao, D.; Guo, H.; Saavedra, G.; Kim, S. S.; Sun, Y. Evaluation of a new aerosol chemical speciation monitor (ACSM) system at an urban site in Atlanta, GA: The use of capture vaporizer and PM2. 5 inlet. ACS Earth and Space Chemistry 2021, 5 (10), 2565-2576.

(20) Docherty, K. S.; Aiken, A. C.; Huffman, J. A.; Ulbrich, I. M.; DeCarlo, P. F.; Sueper, D.; Worsnop, D. R.; Snyder, D. C.; Peltier, R.; Weber, R. The 2005 Study of Organic Aerosols at Riverside (SOAR-1): instrumental intercomparisons and fine particle composition. Atmospheric Chemistry and Physics 2011, 11 (23), 12387-12420.

(21) Ye, D.; Klein, M.; Mulholland, J. A.; Russell, A. G.; Weber, R.; Edgerton, E. S.; Chang, H. H.; Sarnat, J. A.; Tolbert, P. E.; Ebelt Sarnat, S. Estimating acute cardiovascular effects of ambient PM 2.5 metals. Environmental Health Perspectives 2018, 126 (2), 027007.
